# Genomic screens identify a new phytobacterial microbe-associated molecular pattern and the cognate *Arabidopsis* receptor-like kinase that mediates its immune elicitation

**DOI:** 10.1186/s13059-016-0955-7

**Published:** 2016-05-09

**Authors:** G. Adam Mott, Shalabh Thakur, Elwira Smakowska, Pauline W. Wang, Youssef Belkhadir, Darrell Desveaux, David S. Guttman

**Affiliations:** Department of Cell & Systems Biology, University of Toronto, 25 Willcocks St., Toronto, Ontario Canada; Gregor Mendel Institute (GMI), Austrian Academy of Sciences, Vienna Biocenter (VBC), Dr Bohr Gasse 3, Vienna, 1030 Austria; Centre for the Analysis of Genome Evolution & Function, University of Toronto, Toronto, Ontario Canada

## Abstract

**Background:**

The recognition of microbe-associated molecular patterns during infection is central to the mounting of an effective immune response. In spite of their importance, it remains difficult to identify these molecules and the host receptors required for their perception, ultimately limiting our understanding of the role of these molecules in the evolution of host-pathogen relationships.

**Results:**

We employ a comparative genomics screen to identify six new immune eliciting peptides from the phytopathogenic bacterium *Pseudomonas syringae*. We then perform a reverse genetic screen to identify *Arabidopsis thalian*a leucine-rich repeat receptor-like kinases required for the recognition of these elicitors. We test the six elicitors on 187 receptor-like kinase knock-down insertion lines using a high-throughput peroxidase-based immune assay and identify multiple lines that show decreased immune responses to specific peptides. From this primary screen data, we focused on the interaction between the xup25 peptide from a bacterial xanthine/uracil permease and the Arabidopsis receptor-like kinase xanthine/uracil permease sensing 1; a family XII protein closely related to two well-characterized receptor-like kinases. We show that xup25 treatment increases pathogenesis-related gene induction, callose deposition, seedling growth inhibition, and resistance to virulent bacteria, all in a xanthine/uracil permease sensing 1-dependent manner. Finally, we show that this kinase-like receptor can bind the xup25 peptide directly. These results identify xup25 as a *P. syringae* microbe-associated molecular pattern and xanthine/uracil permease sensing 1 as a receptor-like kinase that detects the xup25 epitope to activate immune responses.

**Conclusions:**

The present study demonstrates an efficient method to identify immune elicitors and the plant receptors responsible for their perception. Further exploration of these molecules will increase our understanding of plant-pathogen interactions and the basis for host specificity.

**Electronic supplementary material:**

The online version of this article (doi:10.1186/s13059-016-0955-7) contains supplementary material, which is available to authorized users.

## Background

Effective immunity in plants relies upon a multi-tiered innate immune recognition system to successfully identify and appropriately respond to microbial invaders [1, 2]. This response requires the ability to quickly detect the presence of potential pathogens, effective mechanisms to disseminate that information through the organism, and finally appropriate physiological responses capable of controlling and clearing infection. The initial threat detection is largely accomplished through the recognition of microbe-associated molecular patterns (MAMPs), which are highly conserved immune elicitors derived from invading microbes. In the case of proteinaceous MAMPs, the genes encoding these microbial signatures are likely required for survival and, therefore, under strong negative selection as a whole [3]. If this were not the case, they would be modified or eliminated through natural selection in order to subvert host recognition. Nevertheless, while the genes encoding MAMPs are under strong negative selection overall, individual residues can show signals of positive selection for diversity [3]. This variation in what are otherwise conserved proteins may help the microbe avoid or dampen host recognition. In fact, MAMP sequence diversity has been shown to be associated with variation in the intensity of the immune response elicited by MAMP peptides [4–6].

The recognition of MAMPs is mediated through direct binding to a pattern-recognition receptor (PRR) on the plant cell surface. Characterized plant PRRs of peptide MAMPs contain an extracellular domain with a number of leucine-rich repeat (LRR) domains responsible for MAMP binding and can be divided based on their intracellular moieties into receptor-like kinases (RLK) and the closely related receptor-like proteins (RLP), which lack an intracellular kinase domain [7, 8]. These two gene families in *Arabidopsis thaliana* have 223 and 57 LRR-containing members, respectively [9–11]; a level of genetic diversification suggestive of the key role these proteins play in plant evolutionary success. Immune signaling requires PRRs to work in complex with regulatory co-receptors, such as the Brassinosteroid Insensitive1-associated receptor kinase1 (BAK1) that regulates LRR-containing PRR activation [12, 13]. This protein is an LRR-RLK that interacts with the PRR and appears to help shape the receptor pocket that binds directly to the MAMP [12, 13]. While this co-receptor is not required for PRR-MAMP binding, it does participate in MAMP binding and both of these LRR-RLKs are required for full activation of immune-associated receptor-like cytoplasmic kinases that transduce the immune activation signal.

The importance of MAMPs to plant immunity has led to considerable interest in their identification, yet surprisingly few MAMPs have been characterized. In fact, the majority of work on proteinaceous MAMPs to date has focused on flagellin and elongation factor Tu (EF-Tu) and their respective eliciting peptides flg22 and elf18 [5, 6]. This is due in no small part to the technical challenges involved in the identification of both MAMPs and the plant components required for their perception using traditional means. More recently, with the proliferation of high-throughput sequencing and advances in bioinformatic techniques, predictive methods for MAMP identification have arisen. We have previously described such an approach based on the unique evolutionary signature of MAMP peptides that arises due to strong negative pressure to maintain protein function coupled to positive pressure to mutate sites in order to avoid plant perception [3]. While this early demonstration successfully identified peptides that elicited an immune response, it was not aimed at identifying the cognate host receptors required for recognition of these elicitors and consequently only addressed half of the issue. Another predictive approach has also recently been used to identify novel damage-associated molecular patterns (DAMPs) using sequence homology to known DAMPs [14]. Such approaches will become even more powerful as more genomes are sequenced and more immune elicitors are identified. Nevertheless, the host signaling components required for perception of these novel elicitors have yet to be identified.

The present study couples our computational pipeline for elicitor identification to a reverse genetic screen for cognate receptors using a high-throughput peroxidase assay. We show here both the identification of a novel *Pseudomonas syringae* MAMP that stimulates defence gene expression, induces callose deposition *in planta*, causes seedling growth inhibition, and functionally protects the plant from subsequent pathogen challenge, as well as the identification of an *Arabidopsis thaliana* LRR-RLK that binds the peptide and is required for the perception of this novel MAMP.

## Results

### Computational identification of immune elicitors

The screen for novel peptide elicitors was performed by examining *P. syringae* genomes for genes that show an overall pattern of strong negative selection for maintenance of required protein function, coupled to localized strong positive selection for avoidance of plant perception. We have used this approach previously to identify immune elicitors in *P. syringae* [3], but elected to generate a new set of candidates for this study due to the availability of vastly more genome sequences, which provided much more predictive power (see “Discussion” for more details). We examined 54 *P. syringae* isolates (Additional file 1: Table S1) to identify 3157 ortholog core gene families, as defined by being present in at least 90 % of the isolates. A PAML selection analysis of this core gene set identified 172 genes that had at least one positively selected residue with ω ratio significantly greater than 1.0, and which rejected the neutral and negative selection null model (M7) in favor of the positive selection model (M8). Our previous study found that many putative immune elicitors had clusters of positively selected residues [3], therefore we used the Bayes Empirical Bayes estimates from the M8 model to narrow the list to those with clusters of at least three positively selected sites in a window of 25 residues, producing a candidate pool of 61 genes (Additional file 1: Table S2). This candidate pool included the well-established MAMPs EF-Tu and flagellin, ranking fifth and ninth, respectively, based on the number of predicted positively selected sites.

From the list of 61 positively selected core genes we identified a short-list of candidate peptides for this study based on: the level of significance of the PAML analysis; the overall strength of positive selection for the sequence; the number of positively selected residues clustered within a 25 residue window; the strength of selection acting on these clustered residues; and the likelihood of a false-positive signal due to the underlying alignment and proximity to the end of an assembly contig (see “Methods” for more details). We identified seven candidate 25-amino-acid peptides for analysis, but later dropped one from the study due to inconsistent responses (Table 1 and Additional file 1: Table S3).

**Table 1:** Predicted peptide MAMPs

Peptide name	Gene description	NCBI Gene ID	*P* value^a^	PSS (#)^b^
xup25	Xanthine/uracil permease family protein	1182392	1.13E-03	5
hyp25	Conserved hypothetical protein	1182322	1.60E-06	12
isp25	4-diphosphocytidyl-2C-methyl-D-erythritol kinase	1182741	3.13E-03	8
atp25	ABC transporter, permease protein	1182995	8.46E-03	6
omp25	Outer membrane protein	1183357	2.91E-04	8
mpp25	Membrane protein, putative	1185283	1.99E-05	6

^a^ *P* value rejecting null hypothesis of neutral evolution

^b^Number of positively selected sites

### High-throughput peroxidase immune elicitation assay

We developed a sensitive, high-throughput, microtiter-plate based screening assay based on innate immunity-induced peroxidase (POX) activity as a quantitative measure of immune function in order to efficiently screen large numbers of candidate elicitor peptides and LRR-RLK T-DNA insertion lines. The apoplastic POX enzymes play a role in the plant response to both wounding and pathogen infection [15–17]. These two responses, however, are temporally distinct, a fact that we used to avoid confounding our assay with wound-induced POX activity caused during tissue harvest. While POX activity has been used previously to study the effects of MAMP treatment [5], this is its first adaptation to a high-throughput screen using intact plant tissue. Importantly, the assay also showed excellent responses at a variety of doses of flg22, with sensitivity similar to the traditional ROS assay (Additional file 2: Figure S1).

**Fig. 1:**
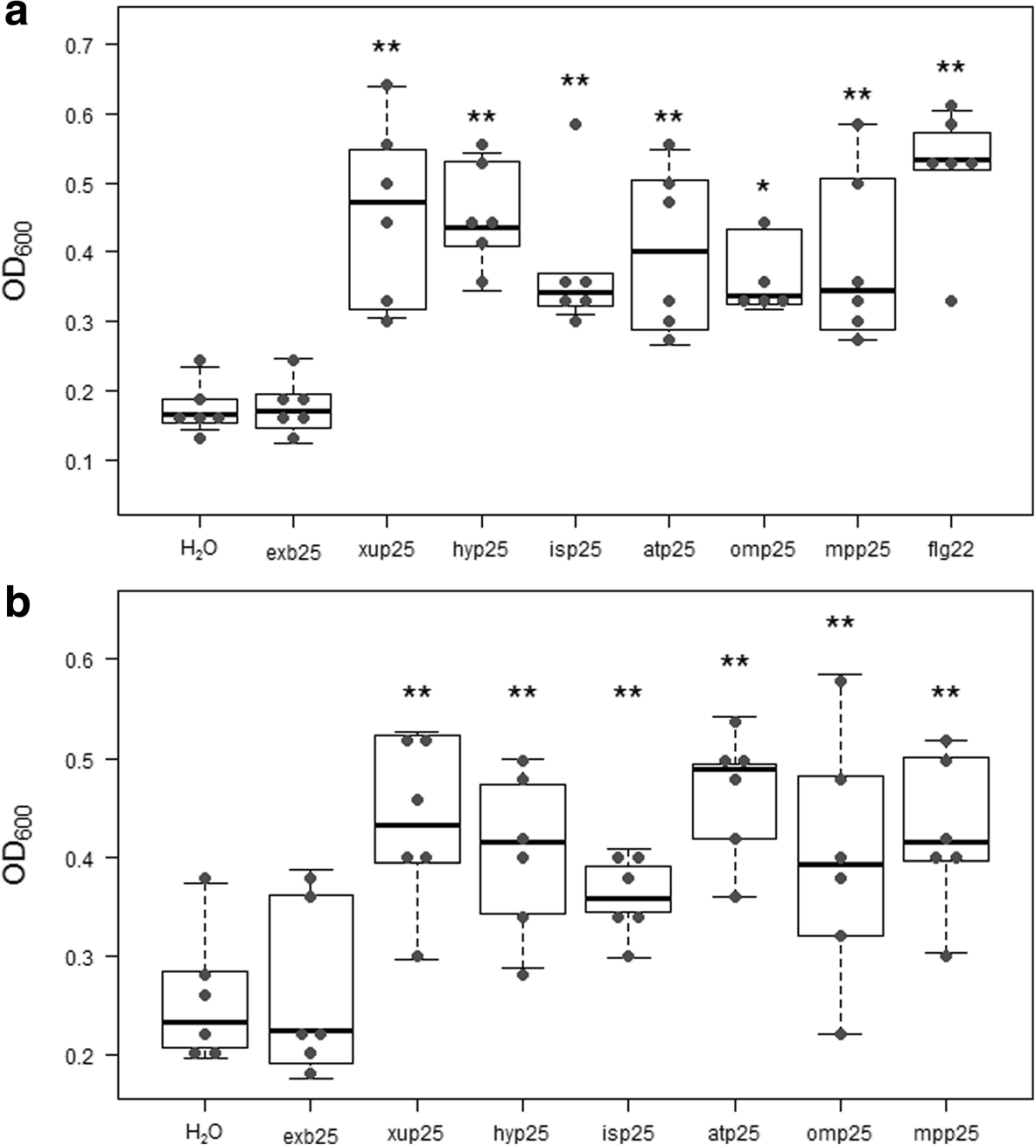
MAMP treatment causes increased POX activity. Leaf disks from *A. thaliana* ecotype Col-0 (**a**) or Ws (**b**) plants were treated with water or 1 μM of peptide and total POX activity was measured 20 h after treatment. *Graphs* are data from a single representative experiment (n = 6, *P <0.05, **P <0.01, ***P <0.001, pairwise paired Student’s *t*-test vs. the water control, corrected with Holm-Bonferroni). *Closed circles* represent individual observations, the *boxes* show the first quartile value, median value, and third quartile value, while the *whiskers* extend to the lowest and highest values in the data that are not deemed to be outliers (i.e. they are within 1.5 * IQR of the quartile value). All experiments were conducted a minimum of seven times with similar results

The six candidate elicitors listed in Table 1 were tested for their ability to stimulate POX activity, and compared to the known MAMP flg22, a water-treated control, and a negative treatment consisting of an equimolar solution of a 25 amino acid peptide taken from a *P. syringae* gene present in the core genome that shows no evidence of positive selection. The four negative control peptides used are listed in Additional file 1: Table S4 and one member of the group was randomly assigned to each replicate experiment. These negative control peptides showed no immune elicitation *in planta* (Additional file 3: Figure S2) and are shown as a pooled single dataset for clarity where appropriate (these pooled data are marked as neg25). The six candidate peptides all elicited POX expression above the baseline (water control) and the neg25 negative control peptide level, although they were generally weaker elicitors than flg22 in *A. thaliana* ecotype Col-0 plants (Fig. 1a). This level of elicitation for peptide elicitors is not unexpected, as flg22 is widely reported to be unique in the strength of its immune elicitation when compared to other peptide MAMPs and DAMPs [3, 14]. We were, however, concerned with the possibility that our observed activity may be due to contaminating flg22 in our peptide preparations. In order to exclude this possibility, we also performed the POX assays in the *A. thaliana* ecotype Wassilewskija (WS) (Fig. 1b), which is insensitive to flg22 (Additional file 4: Figure S3) [18]. The activity of our peptides was maintained in WS, confirming that the activity arises from the novel peptide elicitors and is not due to contaminating flg22 peptide.

### Reverse genetic screen to identify MAMP receptors/co-receptors

**Fig. 2:**
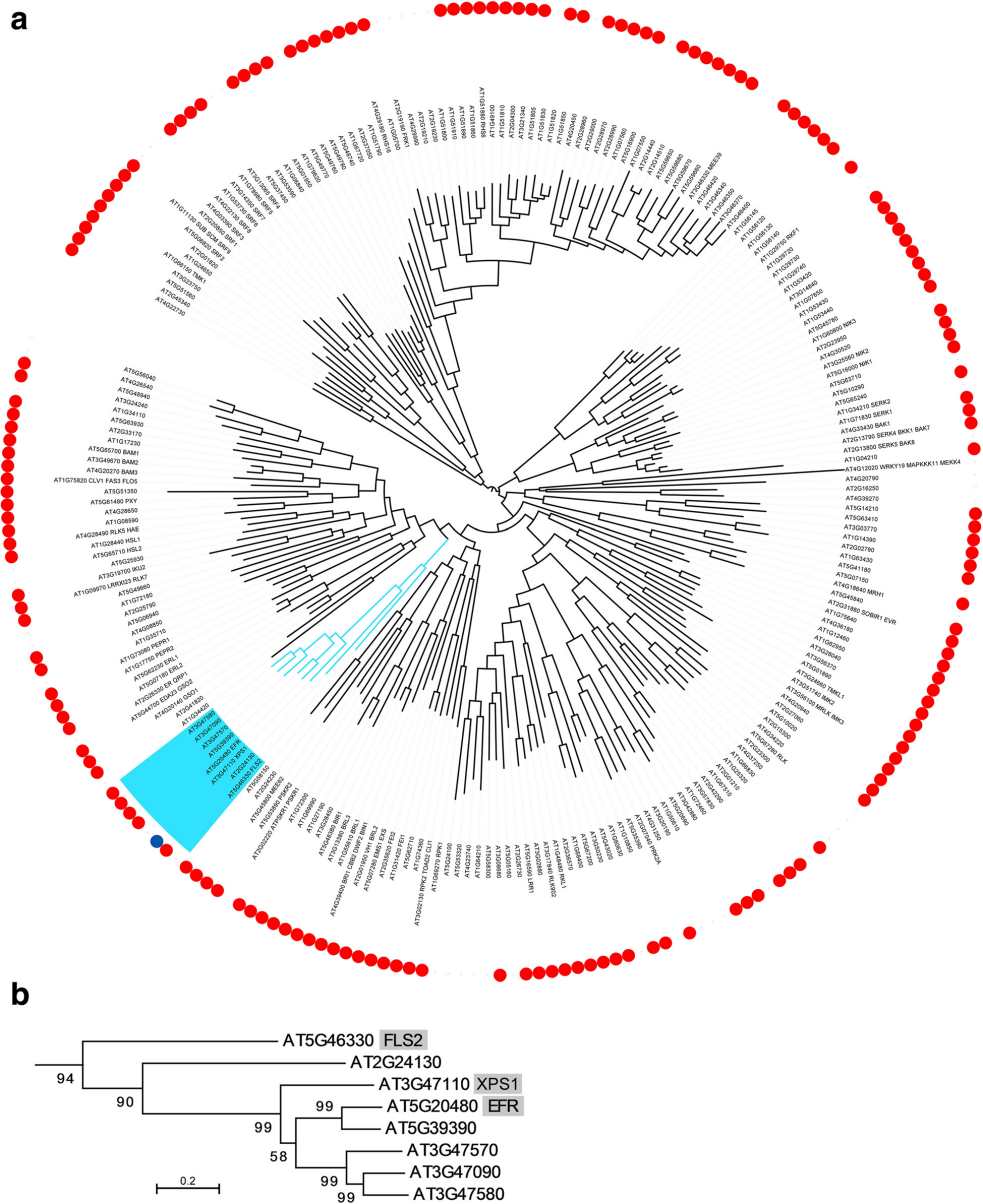
*Arabidopsis thaliana* PRRs form a single phylogenetic cluster within a maximum likelihood phylogeny of LRR-RLKs. **a** Maximum likelihood phylogenetic analysis of *A. thaliana* LRR-RLKs. A total of 187 T-DNA lines, representing 169 genes (*red circles*) were screened for response to the six candidate elicitors. The clade containing FLS2, EFR, and XPS1 (*blue circle*) is highlighted in *blue*. **b** Maximum likelihood phylogenetic analysis of *A. thaliana* LRR-RLKs in the XII family, which contains XPS1. This clade includes FLS2 and EFR, which recognize flagellin (flg22 peptide MAMP) and EF-Tu (elf18 peptide MAMP), respectively

Having identified a set of elicitors that induced a robust and reproducible immune response in *A. thaliana* ecotypes Col-0 and WS using the POX assay, we set out to identify plant LRR-RLKs required for the perception of these peptides. We identified a candidate list of *A. thaliana* ecotype Col-0 LRR-RLKs by searching the *A. thaliana* protein database for all proteins containing both an LRR and a kinase domain; recovering 227 proteins containing both domains, of which 216 also contain predicted transmembrane domains (Additional file 1: Table S5 and Fig. 2a, similar to previously published studies [9, 11]). We obtained 187 T-DNA insertional mutants representing 169 of the LRR-RLK encoding genes (several lines had T-DNA insertions in the same gene; Fig. 2a).

We performed a primary screen on the 187 T-DNA LRR-RLK insertion lines for their ability to respond to our six candidate elicitors using the high-throughput POX assay. Each assay involved testing six replicate plants per genotype, with ten leaf cores per plant. All controls and peptides were assayed in parallel. Overall, the 187 T-DNA lines were assayed 3513 times (excluding negative controls), with a median of 496 assays per elicitor, 16.5 assays per T-DNA line, and 2.7 assays per elicitor per line.

**Fig. 3:**
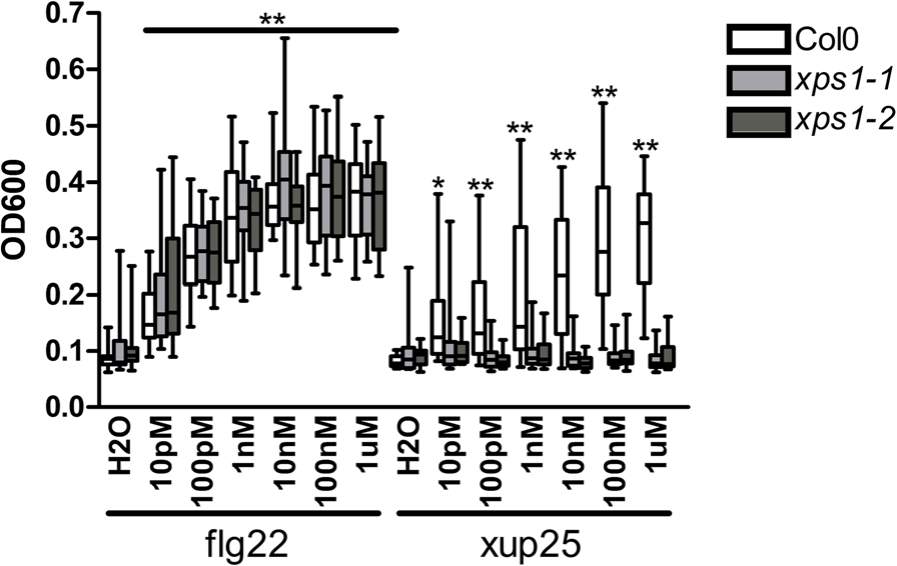
XPS1 is required for POX induction by xup25. Leaf disks from *A. thaliana* ecotype Col-0 (*white boxes*), *xps1-1* (*light gray boxes*), or *xps1-2* (*dark grey boxes*) plants were treated with water or the noted concentration of flg22 or xup25 peptide and total POX activity was measured 20 h after treatment. *Boxes* show the lower quartile value, median value, and upper quartile value, while the *whiskers* extend to the lowest and highest values. Statistical comparisons are made between each treated sample and the untreated control of the same genotype (n = 18, *P <0.05, **P <0.01, repeated measures ANOVA followed by Dunnett’s Test)

Our aim for this study was to identify an LRR-RLK that was specifically required for mounting an immune response against a specific elicitor. We therefore focused on LRR-RLK T-DNA lines that were unable to respond to a single peptide, but which retained their ability to respond to flg22 and the other elicitors. Of the T-DNA lines that met this criterion, a line with an insertion in locus At3G47110 (hereafter *XPS1*) was of particular interest. XPS1 is evolutionarily closely related to the two best characterized peptide sensing LRR-RLKs: EFR and FLS2 [19, 20], as well as the *A. thaliana* ortholog of the rice LRR-RLK XA21, which recognizes a sulphated peptide from the RaxX protein of *Xanthomonas oryzae* pv. *oryzae* [21–24]. The clade containing XPS1, EFR, and FLS2 includes eight LRR-RLKs that form part of LRR-RLK family XII (Fig. 2b) [11]. Importantly, two independent *xps1* T-DNA lines responded normally to flg22 and the five other elicitors, while specifically losing sensitivity to xup25 (Fig. 3, Additional file 5: Figure S4, and Additional file 6: Figure S5). That *xps1* retained flg22 sensitivity equal to that observed in Col-0 also reinforces that the original observed effect was not due to flg22 contamination of the xup25 peptide (Fig. 3).

### The XPS1 LRR-RLK is required for xup25-induced immunity

**Fig. 4:**
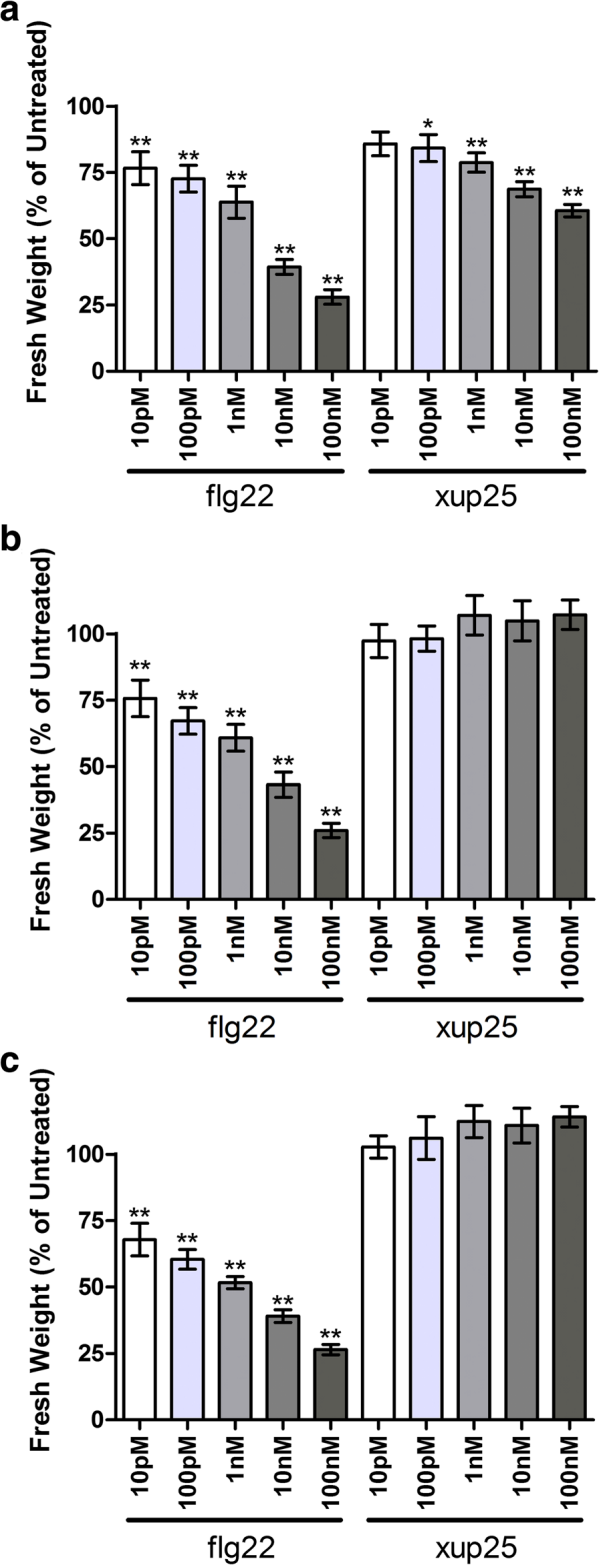
xup25 inhibits seedling growth in an XPS1-dependent manner. Five-day-old seedlings of *A. thaliana* ecotype (**a**) Col-0, (**b**) *xps1-1*, or (**c**) *xps1-2* were grown for a further 10–14 days in liquid MS media containing water or the noted dose of peptide. The seedlings were removed, briefly dried, weighed (fresh weight), and those weights normalized to the mean weight of the control sample. As least four seedlings of each treatment were included and the entire experiment replicated at least three times and all data are presented. Statistical comparisons are made between each treated sample and the untreated control of the same genotype (n ≥ 12, *P <0.05, **P <0.01, one-way ANOVA followed by Dunnett’s Test)

MAMP treatment of *A. thaliana* results in many well-characterized responses, including inhibition of seedling growth [25], induction of defence gene expression [26], physiological changes including reinforcing the cell wall through callose deposition [25], and increased pathogen resistance [27]. To confirm our primary screen results, we first tested for the ability of xup25 to inhibit seedling growth in an XPS-dependent manner, a response that has been associated with immune activation [25]. We treated Col-0 and *xps1* seedlings with flg22 and xup25 in liquid media, measured their subsequent growth, and found that the growth of Col-0 seedlings was significantly inhibited by both flg22 and xup25 peptides in a dose-dependent manner, while *xps1* seedlings were growth-inhibited only by the flg22 peptide (Fig. 4).

**Fig. 5:**
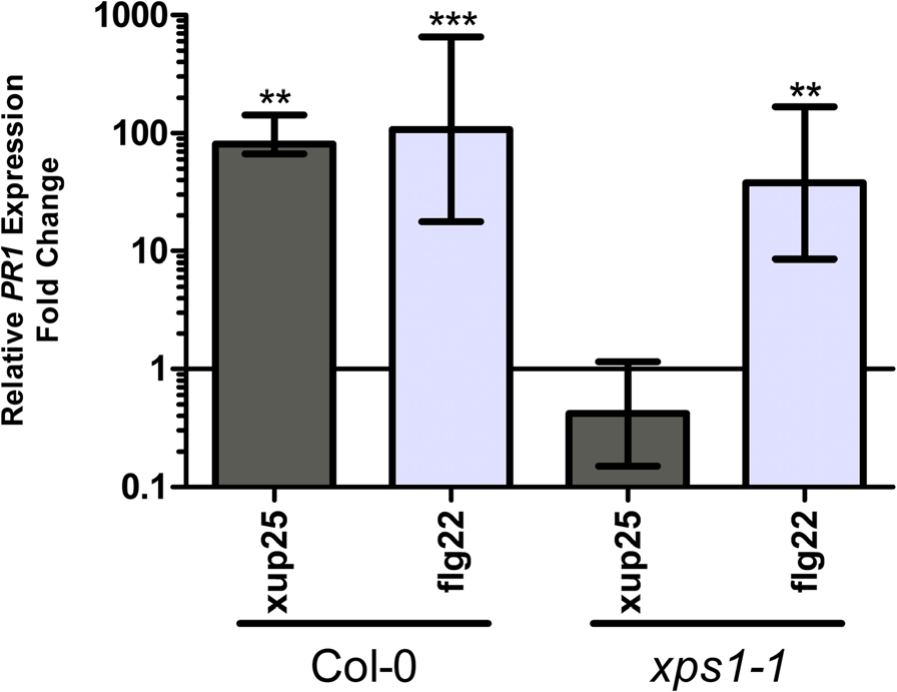
xup25 induces defence gene expression in an XPS1-dependent manner. Quantitative real-time PCR analysis of the relative expression of the defence gene *PR1* in Col-0 and *xps1-1 A. thaliana* plants after 24 h of treatment with water or 10 μM of peptide normalized to UBQ10 expression. Tissue from two individuals for each treatment were pooled prior to RNA harvest. Bars represent mean values +/- s.e. from three biological experiments each assayed in triplicate. Statistical analysis was carried out on dCt values, comparing treatments to water treated control samples using pairwise student’s t-tests with Holm-Bonferroni correction (**P <0.01, ***P <0.001). No significant difference was observed between Col-0 and *xps1-1* plants treated with flg22

The defence gene *PR1* has been used extensively as a general marker of immune stimulation [25, 26]. We tested relative *PR1* expression by treating Col-0 and *xps1* plants with flg22 and xup25. Col-0 plants showed strong *PR1* induction to both treatments relative to the water control after 24 h, while the *xps1* line responded only to the flg22 treatment (Fig. 5).

**Fig. 6:**
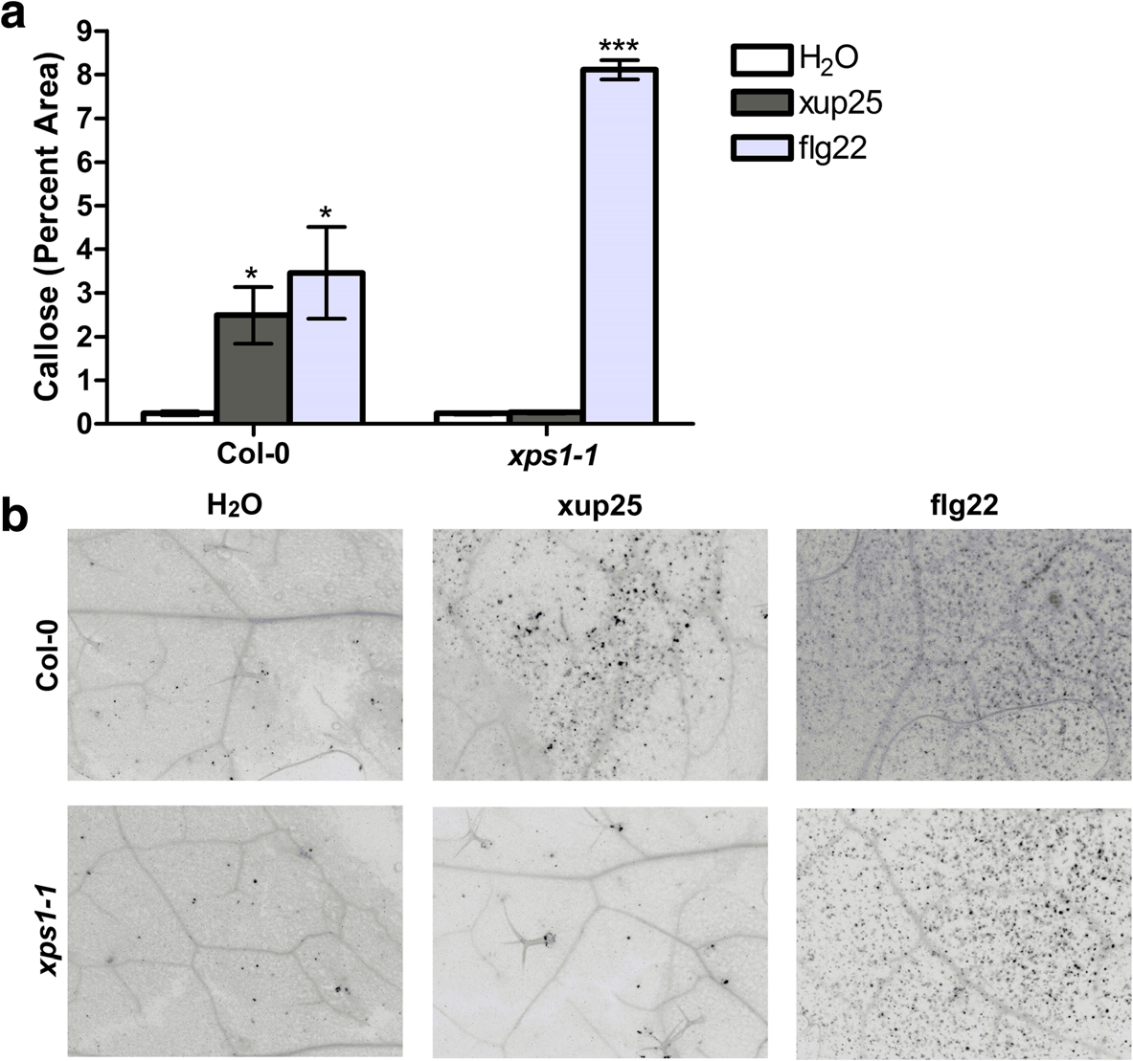
xup25-induced callose deposition is XPS1-dependent. Leaves from *A. thaliana* ecotype Col-0 or *xps1-1* plants were pressure infiltrated with water or 10 μM of peptide. After 24 h of treatment the leaves were harvested, cleared, and callose deposits were stained prior to epifluorescent microscopy. **a** The proportion of the image with callose present was determined and data from a single representative trial is shown (n = 6, *P <0.05, ***P <0.001, pairwise Student’s *t*-test, corrected with Holm-Bonferroni). **b** Representative images of the callose deposits for each treatment are shown. Callose deposits are indicated by dark spots; the images have been converted to gray scale and inverted. The experiment was repeated four times in total with similar results, for a total n = 26

Another well-characterized marker of MAMP treatment is the deposition of callose to reinforce the plant cell wall [25]. We measured the levels of callose deposition following 24 h of treatment with flg22 or xup25. The treatment of Col-0 plants led to increased callose deposition in response to both peptides, whereas the *xps1* line showed callose deposition when treated with flg22 but not xup25, which showed no difference from the water control (Fig. 6 and Additional file 7: Figure S6).

**Fig. 7:**
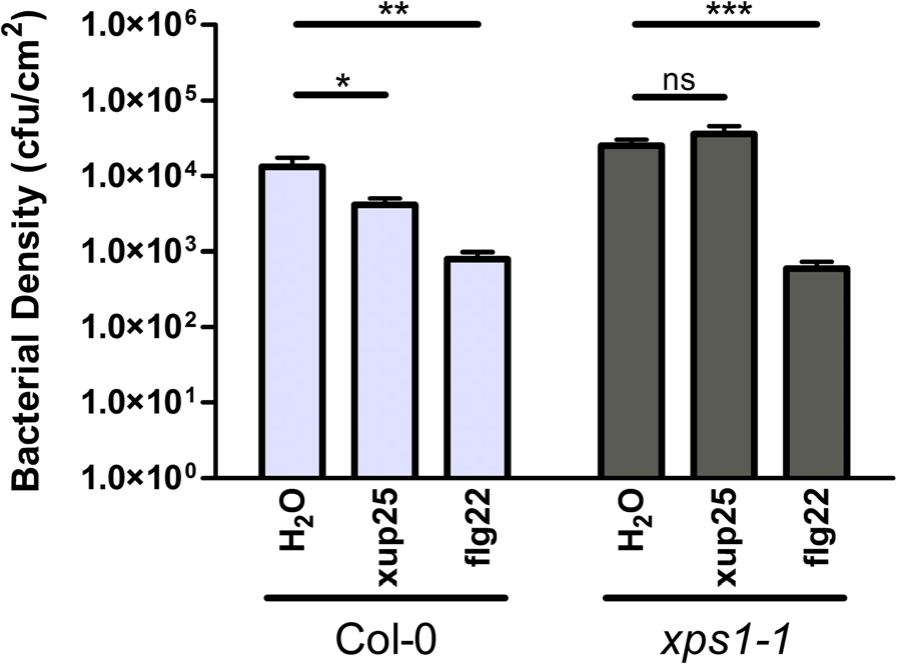
xup25 protects from pathogen challenge in an XPS1-dependent manner. Leaves from *A. thaliana* ecotype Col-0 or *xps1-1* plants were pressure infiltrated with water or 10 μM of peptide 24 h prior to infiltration with *Pto* DC3000 (0.0005 OD). Tissue was harvested after 24 h of *in planta* bacterial growth and lysates were plated on selective media. Data from a single representative experiment is shown (mean +/- s.e., n = 6, *P <0.05, **P <0.01, ***P <0.001, ns not statistically significant, pairwise Student’s *t*-test, corrected with Holm-Bonferroni). Each experiment was repeated three times with similar results. cfu, colony forming units. No significant difference was observed between Col-0 and *xps1-1* plants treated with flg22

The ultimate aim of the immune response is to increase pathogen resistance by suppressing colonization and growth of microbes in plant tissues. To measure elicitor-induced pathogen resistance, and to test the role of XPS1 in suppressing pathogen growth in response to xup25, we performed a virulence suppression assay by pre-treating plants with flg22 or xup25 peptides for 24 h, and then challenging the plants with the highly virulent strain *P. syringae* pv. tomato DC3000 (*Pto* DC3000) via pressure infiltration. Plants that are pre-treated with immune-eliciting peptides should show lower levels of pathogen growth. Both Col-0 and *xps1* plants pre-treated with flg22 significantly suppressed *in planta* growth of *Pto* DC3000 24 h post infection (Fig. 7). In contrast, while xup25 pre-treatment was able to suppress *Pto* DC3000 growth in Col-0, it did not suppress pathogen growth in the *xps1* background, resulting in bacterial densities indistinguishable from the water-treated controls.

### XPS1 specifically binds xup25

**Fig. 8:**
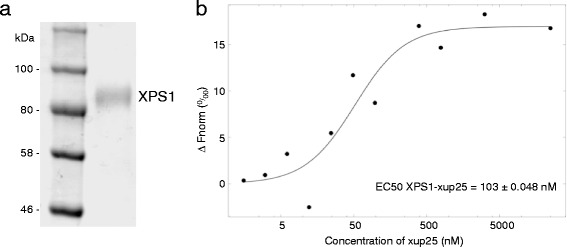
XPS1 binds specifically to xup25. **a** *Coomassie stain* of purified XPS1 ectodomain used in MST assays. **b** Quantification of binding between the XPS1 ectodomain and xup25 by label-free MST. The XPS1 ectodomain was kept at a constant concentration (0.5 μM) whereas varying peptide concentrations were added. *Data points* indicate the difference in normalized fluorescence (%) generated by xup25 binding to the XPS1 ectodomain. *Curves* are plots of xup25 concentrations against percent changes of normalized fluorescence (ΔFnorm [%] *y-axis*). Curve fitting was performed by using the NT affinity analysis software from Nanotemper. The EC50 value calculated at MST power of 60 % is indicated on top. The binding profile is representative of two independent assays performed with two independent protein preparations (see Additional file 9: Figure S8). The standard error of the regression fit is 2.866732

In order to demonstrate that XPS1 and xup25 represent a cognate MAMP-PRR pair, we next tested the binding of the peptide to the ectodomain of the receptor. For this, we expressed the extracellular domain of XPS1 in insect cells and purified it to near homogeneity (Fig. 8a). Subsequently, we used label-free microscale thermophoresis (MST) [28] to study the abilities of xup25 to bind to XPS1. As shown in Fig. 8b, XPS1 was able to bind the xup25 peptide with an observed EC50 of 103 nM ± 0.048 at an MST power of 60 %. We repeatedly performed our measurements on two independent protein preparations to ensure reproducibility. Our second assay at the same MST power displayed a similar binding profile, yet showed an increased EC50 (381 nM ± 0.054). A control experiment in which we tested the ability of XPS1 to bind the flg22 peptide demonstrated the specificity of the xup25-XPS1 interaction (Additional file 8: Figure S7). Thus XPS1 can discriminate unrelated peptides and our binding assays strongly indicate that XPS1 is directly involved in the perception of xup25.

## Discussion

In spite of the important role that MAMPs play in plant immunity, only a small number have been identified, and an even smaller number of proteinaceous MAMPs have been linked to specific LRR-containing receptors. The traditional approaches for MAMP identification rely upon refining complex mixtures of molecules from pathogens, which becomes increasingly difficult when working with MAMPs that have weak biological effects under specific test conditions. In addition, our experimental models can only crudely mimic the complex milieu of microbial commensals, mutualists, and parasites that constantly interact with plants in the real environment. Interactions that we perceive as weak under rarefied laboratory conditions may in fact be extremely important under specific ecological conditions. While these ecological and evolutionary processes normally limit progress, our computational approach to MAMP identification instead takes advantage of these events by mining for the genetic footprints left by these interactions.

This study used comparative genomics analyses to identify peptides carrying genetic signatures consistent with expectations for MAMPs. Six of these immune-eliciting peptides were used in a reverse genetic screen against 187 *A. thaliana* T-DNA insertion lines of putative PRRs. While most of the knockout lines retained their ability to mount an immune response when challenged with the peptides, one line, *xps1*, was unable to mount any measurable immune response to the xup25 peptide. Importantly, the *xps1* line retained its ability to respond to the other peptides, including flg22. Finally, the XPS1 ectodomain is shown to be involved in the direct binding of xup25.

These results indicate that XPS1 can bind to xup25 and is specifically required for perception and, therefore, xup25 and XPS1 can be considered a MAMP and PRR pair in the strictest sense.

The xup25 peptide is derived from a xanthine/uracil permease family protein (conservation is shown in Additional file 9: Figure S8). There is no clear reason why this protein should be a source of a peptide MAMP beyond the fact that it is highly conserved. In fact, all six candidate elicitors identified in this study are broadly conserved among the bacteria, while some are even found in the archaea (data not shown). Four of the candidates, including the protein encoding xup25, are membrane proteins, which while not a requirement for a MAMP (q.v. EF-Tu), may increase the likelihood that the peptides come in contact with host PRRs.

The amplitude of the immune response we observe with the newly identified peptide elicitors is lower than that observed for flg22. Recent work has shown that natural variation within a MAMP sequence can have large effects on the resulting immune response [4, 29], raising the question of whether we would have observed stronger immune responses if we had designed our peptides based on protein sequences from more divergent bacteria with non-host interactions, rather than *P. syringae*. As there has not yet been a broad survey of the relationship between MAMP origin and the strength of immune elicitation, we can only speculate on the matter based on the limited data available. In the best studied case, the flg22 peptide from *Pseudomonas aeruginosa* elicits a stronger immune response in *A. thaliana* than the corresponding peptide from *P. syringae* [30]. This finding may seem logical, as *P. aeruginosa* is a non-host species rarely found on foliar surfaces, suggesting flg22 may be at least partially responsible for this niche restriction. Upon closer examination, this theory is contradicted by the fact that the flg22 peptide does not elicit an efficient immune response in all non-host plant species studied (e.g. celery [29]), nor do all natural accessions of *A. thaliana* carry the FLS2 PRR that recognizes flg22 [18]. In fact, the opposite argument could also be made, namely that MAMPs from pathogens of a specific plant host should elicit a strong immune response, since these are ecologically relevant interactions. This argument is supported by the observation that FLS2 restricts the growth of even highly virulent pathogens such as *Pto* DC3000 [31]. While type III secreted effectors also strongly influence disease and immunity, these proteins are highly variable even among closely related strains [32–34] and, consequently, likely mediate a relatively small number of all plant-microbe interactions. We elected to test candidate elicitor peptides derived from the host strain *Pto* DC3000, as well as a number of homologous peptides derived from the non-host strain *P. syringae* pv. *phaseolicola* 1448a, but found no significant differential response (data not shown). Future work examining homologous peptides from a wider range of species will help inform which of these two positions is more generally correct.

Previous work in our group has used a similar bioinformatics approach to identifying immune eliciting peptides [3], but the current report represents a significant improvement in several respects. Most notably, the previous work was performed when only three *P. syringae* isolates had been sequenced, necessitating the inclusion of three strains of *Xanthomonas campestris* to achieve sufficient sample size for the selection analyses. The inclusion of these divergent genomes strongly influenced the prediction of the set of core genes. Additionally, the use of two quite divergent species groups negatively influenced the quality of the alignments and ultimately the predictions of positive and negative selection as gap positions are ignored in PAML selection analyses. In the earlier report, we characterized the immune eliciting properties of eight bioinformatically predicted peptides. The proteins from which these peptides were derived were also analyzed as part of this report, however the use of expanded genome data from 54 *P. syringae* strains resulted in our decision to select new candidates for a variety of reasons. Three of the previously identified proteins (PSPTO2757, PSPTO3620, and PSPTO4436) were not present in a sufficient number of strains to be considered part of the core genome in this report. This observation may be true or simply an artefact due to the extensive use of incomplete draft genomes in the current study. Of the remaining five candidates from the previous report, four had positively selected sites (PSPTO3468, PSPTO0175, PSPTO5086, and PSPTO0624), while the last did not show a significant signal of positive selection in the new analysis (PSPTO1253). The inclusion of PSPTO1253 was unique in the previous report as it was based on the presence of a single PSS, making it the least robust prediction of the set. We elected not to use the four previously identified peptides that were also present in this new study since the expanded dataset and subsequent refined analysis identified other candidates with stronger signals based on the number and clustering of positively selected sites. The current analysis of 54 *P. syringae* genomes provides better sequence alignments, a much more reliable prediction of the species-specific core genome, greatly enhanced statistical power to detect signatures of positive selection, and ultimately a more robust prediction of MAMP candidates.

XPS1 is notable as it forms a part of the LRR-RLK family XII along with FLS2 and EFR, the receptors that recognize the peptide MAMPs flg22 and elf18, respectively [19, 20]. This clade also contains the *A. thaliana* ortholog of the rice LRR-RLK XA21 (based on reciprocal BLAST analysis), which recognizes a sulphated peptide from the RaxX protein to confer resistance to *Xanthomonas oryzae* pv. *oryzae* [21, 22, 24]. In addition to the phylogenetic proximity, the presence of a predicted 23 LRR domains (data not shown) in the XPS1 extracellular domain suggests the protein acts as a receptor. It has previously been suggested [35] that the number of LRR domains present in a given LRR-RLK can be used to predict whether that protein functions as a co-receptor, such as BAK1 with only five LRR domains [36, 37], or receptor, such as FLS2 or EFR which have 28 and 21 LRR domains, respectively [19, 20]. These observations suggest that the clade of LRR-RLKs containing FLS2, EFR, and XPS1 may be involved generally in MAMP perception and provides several excellent candidates for further inquiry.

There are few previous examples of studies that quantitate the binding affinity between a PRR and its peptide MAMP. While the techniques used in those reports differ from those used here, they generally report IC_50_ values in the low nM range [18, 20, 38]. While not directly comparable, the apparent EC50 of XPS1 for xup25 is in the high nM range. Our attempts to fit our data using a dissociation constant (K_D_) model showed highly significant variation between the two assays we performed (using independent protein preparations) even at different MST powers. Therefore, we were not able to calculate an accurate overall K_D_ for the XPS1-xup25 binding. We attribute this variation in K_D_ to non-optimized binding events driven by the heterogeneous N-glycosylation of the XPS1 ectodomain in our expression system (as observed in coomassie stained samples of the purified protein (Fig. 8a)). Alternatively, the variations in binding constants might reflect the use of a non-optimized xup25 peptide sequence in our binding assays. In this respect, we anticipate that interaction assays involving shorter or longer variants of xup25 will be very informative. Additionally, while we definitively show that the XPS1 binds to xup25 and is required for its biological activity, this does not eliminate the possibility that the peptide binds to other cell surface molecules including co-receptors. A missing required co-receptor in these in vitro binding assays would also explain the observed variability and current efforts in the lab are focused on discovery of other members of the binding complex.

To date, the identification of PRRs has relied primarily on forward genetic screens, with limited examples of successful reverse genetics approaches [14, 20, 39]. While this forward genetics approach has been very successful, it is limited when applied to MAMPs with weak biological effects or less robust phenotypes. We instead employed a reverse genetics approach to screen the predicted elicitors against a collection of predicted *A. thaliana* immune receptors. This method required a collection of LRR-RLK knockout lines and the use of a high-throughput assay to measure immune elicitation (the POX assay), but enabled us to screen a wide diversity of candidate immune receptors. While most LRR-RLK T-DNA lines showed a wild-type response to all tested peptides, some displayed general hyper- or hypo-sensitivity to the suite of elicitors, while a smaller group (including *xps1*) showed specific loss of sensitivity to a single peptide; thereby providing a wealth of leads for the dissection of plant immune signaling pathways.

As genomic and transcriptomic data become more available, these bioinformatics approaches to MAMP and PRR identification will become even more powerful. The continued elucidation of the true range of MAMP epitopes and their cognate receptors will not only benefit plant health, but also increase our understanding of the evolution of the plant-pathogen interaction.

## Conclusions

Here we expand on our previous work, utilizing a predictive bioinformatics approach to identify potential MAMPs based on their unique evolutionary signatures, and then use these immune elicitors in a reverse genetic screen to identify their putative receptors or co-receptors. We show that the xup25 peptide derived from a xanthine/uracil permease protein is a novel MAMP with the associated biological effects, including plant growth inhibition, induction of defence gene expression and callose deposition, and the stimulation of a functional immune response sufficient to suppress the growth of a pathogen. Most significantly, xup25 specifically requires the function of the LRR-RLK XPS1 to elicit all of these responses and directly binds to this plant receptor. While we have focused on one particular interaction to validate the methodology, both the computational MAMP screen and the subsequent reverse genetic receptor screen have provided numerous candidates for future study. Of particular interest are those LRR-RLKs that show consistent patterns of hypo- and hyper-sensitivity to multiple elicitors. These proteins may play key roles in regulating the *A. thaliana* basal immune response, and therefore, are potentially important targets for the development of resistant crops.

## Methods

### Plant materials and growth conditions

All plants were grown with a 12-h photoperiod (100–150 μmol/m^2^s) at 21 °C, followed by a 12-h dark period at 20 °C in a Conviron growth room (Conviron). All T-DNA lines were selected using the T-DNA Express database of the Salk Institute Genomic Analysis Laboratory (SIGnAL, http://signal.salk.edu/cgi-bin/tdnaexpress). Preference was given to lines with insertions in coding sequences. The genotypes of lines of interest were confirmed by PCR using primers designed using the T-DNA primer design tool (http://signal.salk.edu/tdnaprimers.2.html).

### Core-genome prediction

The analysis was performed using protein sequences from the genomes of 54 *P. syringae* strains. The dataset included genomes from 28 strains available in public databases and 26 strains sequenced by the University of Toronto Centre for Analysis of Genome Evolution and Function (Additional file 1: Table S1). The ortholog prediction was performed using the OrthoMCL program with protein blast e-value 1e-10 [40]. The protein sequences were predicted as ortholog pairs if they shared at least 70 % identity over 70 % sequence length. OrthoMCL was used to cluster ortholog pairs into the ortholog protein families using a markov chain clustering algorithm. The ortholog families that were present in at least 90 % of the strains were defined as core gene families.

### Selection analysis

The codon-based multiple alignments were constructed with translated protein sequences using translatorX and the MUSCLE multiple alignment program [41, 42]. The protein-based phylogenetic tree was built for each core gene family using the FastTree program [43]. The patterns of natural selection were detected in the predicted core gene families by using the codeml program in the PAML application suite [44]. The prediction of positive selection in the core genes was performed by applying codeml random-site models M7 (neutral selection) and M8 (positive selection) that allow the ω ratio dN/dS to vary among sites. A likelihood ratio test was used to reject the neutral and negative selection null model (M7) in favor of positive selection model (M8) with the chi-square *P* value <0.01 for all candidate core genes. Empirical Bayes estimates from the model M8 giving a posterior probability >0.5 were used to identify positively selected codons.

### Peptides

The predicted peptide MAMPs were designed from the *P. syringae Pto* DC3000 genome sequence as 25 amino acid sequences. The flg22 sequence is taken from the *P. aeruginosa* genome sequence as a 22 amino acid sequence. All peptides were purchased from the Sheldon Biotechnology Centre at McGill University and GenScript.

### Peroxidase assay

Single leaves were excised from six plants per genotype of mature *A. thaliana* plants. From each leaf ten size one leaf cores were taken and washed for 1 h in 1 mL of 1X MS solution with agitation. After washing, leaves were transferred to individual wells of a clear 96-well assay plate avoiding the use of the edge wells to minimize evaporation effects. Each well received 50 μL of 1X MS buffer alone or supplemented with 1 μM of each peptide. Thus each leaf was tested with each treatment, allowing for paired statistical testing. Plates were sealed with parafilm and incubated for 20 h with agitation. The leaf disks were removed and each well received 50 μL of a 1 mg/mL solution of 5-aminosalicylic acid (A79809, Sigma-Aldrich) pH 6.0 with 0.01 % hydrogen peroxide. The reaction was allowed to proceed for 1–3 min and stopped by the addition of 20 μM 2 N NaOH prior to reading the OD_600_ on a POLARstar OPTIMA microplate reader (BMG Labtech).

### LRR-RLK prediction and alignment

RLKs were identified by identifying all proteins with both an LRR domain similar to the LRR domain of either FLS2 or EFR and a kinase domain similar to the kinase domain of FLS2. Similarity searches were performed using both Delta BLAST [45] and PSI-BLAST [46] with the respective conserved domain. The *A. thaliana* RefSeq database was queried using a minimum E-value threshold of 1e-08, and the searches were iterated until no new sequences were added. RLK candidate proteins were selected for further analysis only if they had a match to both an LRR domain and a kinase domain, resulting in 227 candidates. Global multiple sequence alignment was performed with the MAFFT E-INS-i algorithm [47] and a maximum likelihood phylogeny was constructed in MEGA v5 [48] using partial deletion and 25 % site coverage cutoff, the WAG with Freqs. (+F) substitution mode, gamma distribution with five rate categories, and 100 bootstraps.

### Confirmation of T-DNA insertion lines

The SALK lines SALK_101647 (*xps1-1*) and SALK_101668 (*xps1-2*) were obtained from the Arabidopsis Biological Resource Center (ABRC) [49]. RT-PCR analyses were performed to verify alterations in transcript levels. Plant RNA was extracted from leaves, which were excised, pooled, and flash frozen in liquid nitrogen prior to homogenization by mortar and pestle under liquid nitrogen. Total RNA was extracted using TRI Reagent as per manufacturer’s instructions (Sigma-Aldrich), followed by DNaseI treatment (Thermo Scientific). A total of 2 μg of RNA was used for cDNA synthesis according to the manufacturer’s instructions with the SuperScript II reverse transcriptase (Life Technologies). Amplification of UBQ10 served as an internal control in RT-PCR assays. The PCR primers used were: *XPS1* LRR domain 5’- TCTTCACTAATATTCCTG-3’ and 5’- TGATAGTTTATTGTATGA-3’, *UBQ10* (At4g05320) 5’-CACACTCCACTTGGTCTTGCGT-3’ and 5’-TGGTCTTTCCGGTGAGAGTCTTCA-3’. The PCR included 35 cycles for *XPS1* LRR domain, and 32 cycles for *UBQ10*.

### Real-time PCR assay

A single leaf on two individual plants was pressure infiltrated with water alone or supplemented with 10 μM peptide. After 24 h of treatment the leaves were excised, pooled, and flash frozen in liquid nitrogen prior to homogenization by mortar and pestle under liquid nitrogen. Total RNA was extracted using TRI Reagent as per manufacturer’s instructions (Sigma-Aldrich), followed by DNaseI treatment (Thermo Scientific). A total of 2 μg of RNA was used for cDNA synthesis according to the manufacturer’s instructions with the SuperScript II reverse transcriptase (Life Technologies). The PCR was carried out in a final reaction volume of 20 μL using Maxima SYBR Green/ROX qPCR Master Mix (Thermo Scientific) using the recommended conditions on a BioRad C1000 thermal cycler (BioRad). The PCR primers used were: *PR1* (At2g14610) 5’-CGGAGCTACGCAGAACAACT-3’ and 5’-CTCGCTAACCCACATGTTCA-3’, *UBQ10* (At4g05320) 5’-CACACTCCACTTGGTCTTGCGT-3’ and 5’-TGGTCTTTCCGGTGAGAGTCTTCA-3’. The PCR program used for all reactions was: 95 °C for 10 min, followed by 40 cycles consisting of 95 °C for 15 s, 55 °C for 30 s, and 72 °C for 30 s. Each sample was assayed in triplicate and data were evaluated using BioRad CFX manager software, version 1.6 (BioRad). The relative *PR1* expression was normalized using the *UBQ10* control and reported as fold increase in expression relative to the water treated control using the ΔΔCt method [50].

### Callose assay

*A. thaliana* plants were pressure infiltrated with water alone, or water containing 10 μM or 100 nM of peptide. The leaves were harvested after 24 h of treatment and cleared with lactophenol. The callose deposits were stained with aniline blue and imaged by epifluorescent microscopy. The images were analyzed with ImageJ [51] software and the percentage of callose coverage in each image was calculated excluding staining of trichomes and the vasculature. A minimum of two images were taken from each of two leaves from two plants per treatment. The experiment was replicated four times with similar results.

### Seedling growth inhibition assay

Seedlings of *A. thaliana* ecotype Col-0, *xps1-1*, or *xps1-2* were grown for 5 days on MS-Agar plates prior to transfer of two seedlings to each well of a 24-well plate containing 400 μL of 0.5× MS medium with 1 % sucrose. The seedlings were treated with water or the noted dose of peptide and grown for a further 10–14 days. The seedlings were removed, briefly dried, and weighed (fresh weight). A minimum of four plants per genotype per treatment were included, and the experiment was replicated three times with similar results; all data are presented. For each replicate the mean fresh weight of the water control seedlings was calculated and used to normalize the weights of all seedlings of that genotype as a percentage of the control value.

### Virulence suppression assay

*A. thaliana* plants were pressure infiltrated with water alone or water containing 10 μM of peptide 24 h prior to infection with *Pto* DC3000 (0.0005 OD_600_). Bacterial growth was measured after 24 h. Briefly, surface sterilized leaf disks were homogenized and the resulting culture was serially diluted and plated on solid selective media. The resulting colony counts were used to determine the number of colony forming units per surface area of the leaf. Each treatment was conducted on four leaves per plant of six individual plants. The experiment was repeated three times with similar results.

### XPS1 extracellular domain expression and purification

For XPS1 expression in Hi5 cells, the ectodomain of XPS1 (36-650) was inserted into the baculovirus transfer vector pMelBac B (Invitrogen). The extracellular domain of XPS1 (amino acids 36-650) was cloned by ligation independent cloning between the existing Honey bee melettin signal sequence and the C-terminal Strep II-9x His tag. All clones were verified by Sanger Sequencing. Primers (Forward: GGT CGT ATA CAT TTC TTA CAT CTA TGC GAC GGA GGA GAC TGA TAA ACA AGC ATT GC; Reverse: GCA CCC TGG AAG TAC AGG TTC TCT GAT GAA TGC CTT CTT GGT AAT TCA ACA GAG C) were designed to have an amplification part homologous to the desired boundaries of XPS1 ectodomain and extensions for RecA-mediated SLIC strategy. The StrepII-9x His fused XPS1 ectodomain was produced by secreted expression in baculovirus-infected insect cells, harvested 72 h post infection and purified by Ni-NTA affinity chromatography (Qiagen). The samples were processed twice with a Superdex 200 16/60 column (GE Healthcare) pre-equilibrated with 10 mM Bis-Tris pH 6.0, 150 mM NaCl. Protein purity was checked by SDS-PAGE. The identity of the XPS1 ectodomain was further confirmed by anti-His immunoblots and mass spectrometry.

### MST binding assays

The XPS1 ectodomain was kept at a constant concentration (0.5 μM) in a buffer containing 10 mM Bis-Tris pH6, 150 mM NaCl, 5 % glycerol and 0.01 % Tween, whereas varying peptide concentrations were added. Approximately 4–6 μL of each sample was loaded in a fused silica capillary (NanoTemper Technologies). Measurements were performed at room temperature in a Monolith NT.label free instrument at a constant LED power of 20 and MST power of 60 %. Measurements were performed repeatedly on two independent protein preparations to ensure reproducibility. The data were analyzed by plotting xup25 concentrations against percent changes of normalized fluorescence (ΔFnorm [%] y-axis). Curve fitting was performed by using the NT affinity analysis software from Nanotemper.

### Luminol-based ROS generation assay

Measurement of ROS production was performed using a modified version of the luminol-based assay as described [5]. Ten leaf discs (diameter 4 mm) were taken from the leaves of 4-week-old Col-0 *A. thaliana*. Leaf disks were washed with 200 μL of sterile water for 20 h. The water was removed and replaced with 100 mM Tris-HCl (pH 8.0) containing 20 μg/mL horseradish peroxidase and 34 μg/mL luminol. Wells were treated with water control or the noted dose of peptide. The luminescence was summed over a 2-s interval every 2 min for a period of 60 min on an Infinite M1000Pro microplate reader (TECAN Group Ltd.). Each treatment was performed on six wells.

### Sequence alignment and sequence logo

The protein sequence of the xup25 containing xanthine uracil permease gene from each species used was extracted and aligned using MUSCLE [41]. The alignment was uploaded to the Gene Slider module of the Bio-Analytical Resource for Plant Biology (bar.utoronto.ca) to produce the sequence logo.

### Ethics approval

No ethics approval was necessary for this study.

### Data availability

All of the genomic data produced for this study have been submitted to NCBI; the BioProject Accession numbers for these genomes along with all the publicly available genomes used can be found in Additional file 1: Table S1. The RefSeq ID numbers for all LRR-RLK sequences used can be found in Additional file 1: Table S5.

## Additional files

Additional file 1: Contains Supplementary Tables S1–S5. (DOCX 56 kb) Additional file 2: Figure S1. Comparison of peroxidase- and luminol-based PTI assays. Leaf disks from *A. thaliana* ecotype Col-0 plants were treated with water or the noted dose of flg22 peptide. *Graphs* are data from a single representative experiment. **a** Total POX activity was measured 20 h after treatment (n = 6, ****P* <0.001, pairwise Student’s *t*-test, corrected with Holm-Bonferroni). **b** ROS production was quantified using luminol and horseradish peroxidase with luminescence measured for 2 s every 2 min for 60 min and the total summed. (n = 6, ****P* <0.001, pairwise Student’s *t*-test, corrected with Holm-Bonferroni). *Boxes* show the lower quartile value, median value, and upper quartile value, while the *whiskers* extend to the lowest and highest values. (PNG 216 kb) Additional file 3: Figure S2. Negative control peptides without predicted positively selected sites do not cause increased POX activity. Leaf disks from *A. thaliana* ecotype Col-0 plants were treated with water or 1 μM of the indicated negative control peptide and total POX activity was measured 20 h after treatment. The mean value was calculated for each experimental replicate (n = 6) performed and these means plotted as *data points* on the *graph*. Each peptide was tested independently eight or nine times. *Boxplot* layout is as described in Fig. 1. (PNG 3 kb) Additional file 4: Figure S3. flg22 does not cause increased POX activity in Arabidopsis ecotype Ws. Leaf disks from *A. thaliana* ecotype Ws plants were treated with water or 1 μM of flg22 peptide. Total POX activity was measured 20 h after treatment. The experiment was replicated three times for a total n = 18. *Boxplot* layout is as described in Fig. 1. (PNG 3 kb) Additional file 5: Figure S4. xup25 does not induce POX activity in an independent *xps1* T-DNA insertion line: *xps1-2*. Leaf disks from *A. thaliana xps1-2* plants were treated with water or 1 μM of peptide and total POX activity was measured 20 h after treatment. The mean value was calculated for each experimental replicate (n = 6) performed and these means plotted as *data points* on the *graph*. Each peptide was tested a total of four times except xup25 which was tested six times. *Boxplot* layout is as described in Fig. 1. (PNG 4 kb) Additional file 6: Figure S5. Confirmation of T-DNA insertions for two independent *xps1* T-DNA insertion lines. **a** T-DNA insertion position for *xps1-1* and *xps1-2. Boxes* represent exons and *lines* represent introns. **b** RT-PCR analysis of *XPS1* expression in Col-0, *xps1-1*, and *xps1-2* plants, respectively. The ubiquitously expressed *UBQ10* is shown as a control for cDNA concentration. (PNG 51 kb) Additional file 7: Figure S6. xup25 does not induce callose deposition in an independent *xps1* T-DNA insertion line: *xps1-2*. Leaves from *A. thaliana xps1-2* plants were pressure infiltrated with water or 10 μM of xup25, 100 nM xup25, or flg22 peptide. After 24 h of treatment the leaves were harvested, cleared, and callose deposits were stained prior to epifluorescent microscopy. The proportion of the image with callose present was determined (n = 6, **P* <0.05, ***P* <0.01, pairwise Student’s *t*-test, corrected with Holm-Bonferroni). No significant difference was observed between Col-0, *xps1-1*, and *xps1-2* plants treated with flg22. (PNG 242 kb) Additional file 8: Figure S7. XPS1 binds specifically to xup25. Additional replicates of XPS1 binding assays. Titration of increasing amounts of xup25 peptide (**a**), but not flg22 (**b**), to a constant amount of XPS1 ECD (0.5 μM) induces a significant MST signal shift. At each peptide concentration a measurement was made using 60 % MST power and the values used to determine the K_D_ or the EC50 with the NT affinity analysis software from Nanotemper. The K_D_ fit plots are shown and both the K_D_ and EC50 values are noted wherever possible. The calculated EC50 and K_D_ values for xup25 were EC50 of 0.381 μM and K_D_ of 0.111 μM. The flg22 binding data could not be fitted using the EC50 fit and has a K_D_ of 13.13 μM. The standard error of the regression fit is 3.8404566 for (**a**) and 6.4503735 for (**b**). (PNG 20 kb) Additional file 9: Figure S8. Diversity of xup25 sequence across *P. syringae* species. **a** The protein sequences of the xup25 peptide from each species used in this study aligned. **b** The protein sequence of the xanthine uracil permease gene was aligned and used to produce a sequence logo, the xup25 sequence logo is marked and expanded below showing the diversity present in the peptide sequence. (PNG 1736 kb)
