# Supplementary material for: Genomic screens identify a new phytobacterial microbe-associated molecular pattern and the cognate Arabidopsis receptor-like kinase that mediates its immune elicitation
Source: Genome Biol. 2016 May 9;17:98. doi: 10.1186/s13059-016-0955-7 (PMC4862170; doi:10.1186/s13059-016-0955-7)
Supplement: Additional file 1: — Contains Supplementary Tables S1–S5. (DOCX 56 kb) [file 13059_2016_955_MOESM1_ESM.docx]

**Additional file 1**

**Supplemental Material for:**

**Genomic screens identify a new phytobacterial MAMP and the cognate Arabidopsis receptor-like kinase that mediates its immune elicitation**

**G. Adam Mott^1^, Shalabh Thakur^1^, Elwira Smakowska^2^, Pauline W. Wang^3^, Youssef Belkhadir^2^, Darrell Desveaux^1,3*^**^§^**, David S. Guttman^1,3*^**^§^

^1^ Department of Cell & Systems Biology, University of Toronto, 25 Willcocks St., Toronto, Ontario, Canada

^2^ Gregor Mendel Institute (GMI), Austrian Academy of Sciences, Vienna Biocenter (VBC), Dr Bohr Gasse 3, Vienna 1030, Austria

^3^ Centre for the Analysis of Genome Evolution & Function, University of Toronto, Toronto, Ontario, Canada

* These authors contributed equally to this work

^§^ Corresponding authors

Email addresses:

GAM: adam.mott@utoronto.ca

ST: shalabh.thakur@mail.utoronto.ca

ES: elwira.smakowska@gmi.oeaw.ac.at

PWW: pauline.wang@utoronto.ca

YB: youssef.belkhadir@gmi.oeaw.ac.at

DD: darrell.desveaux@utoronto.ca

DSG: david.guttman@utoronto.ca

| **Table S1 – *P. syringae* Strains used for MAMP Prediction** | | | |
| --- | --- | --- | --- |
| **Strain** | **Pathovar** | **Reference** | **NCBI BioProject ID** |
| Pto T1 | tomato | Almeida NF et al., 2009 | PRJNA19697 |
| Pae 0893_23 | aesculi | Baltrus DA et al., 2011 | PRJNA33201 |
| Ptt DSM50252 | aptata | Baltrus DA et al., 2011 | PRJNA181496 |
| Pja M301072 | japonica | Baltrus DA et al., 2011 | PRJNA181490 |
| Pla M301315 | lachrymans | Baltrus DA et al., 2011 | PRJNA33205 |
| Pla M302278 | lachrymans | Baltrus DA et al., 2011 | PRJNA33219 |
| Pma ES4326 | maculicola | Baltrus DA et al., 2011 | PRJNA33215 |
| Pmo 301020 | mori | Baltrus DA et al., 2011 | PRJNA181489 |
| Pmp M302280 | morsprunorum | Baltrus DA et al., 2011 | PRJNA33203 |
| Ppi 1704B | pisi | Baltrus DA et al., 2011 | PRJNA181491 |
| Pta ATCC11528 | tabaci | Baltrus DA et al., 2011 | PRJNA282426 |
| Psy Cit7 | N/A | Baltrus DA, et al. 2011 | PRJNA33213 |
| Pan M302091 | actinidiae | Baltrus DA, et al., 2011 | PRJNA33217 |
| Pto DC3000 | tomato | Buell CR et al., 2003 | PRJNA57967 |
| Pac M302273 | aceris | Baltrus DA, et al. 2011 | PRJNA33225 |
| Psy B728a | syringae | Feil H et al.,2005 | PRJNA57931 |
| Pae NCPPB3681 | aesculi | Green S et al., 2010 | PRJNA39839 |
| Pph 1448A | phaseolicola | Joardar V et al., 2005 | PRJNA12416 |
| Pav BPIC_631 | avellanae | O'Brien et al., 2012 | PRJNA207988 |
| Pav ISPaVe013 | avellanae | O'Brien et al., 2012 | PRJNA84319 |
| Pav ISPaVe037 | avellanae | O'Brien et al., 2012 | PRJNA84321 |
| Pgy B076 | glycinea | Qi M et al., 2011 | PRJNA51517 |
| Psv NCPPB3335 | savastanoi | Rodriguez-Palenzuela P et al. 2010 | PRJNA41887 |
| Pta 6606 | tabaci | Studholme DJ., 2009 | PRJNA165717 |
| Pto K40 | tomato | Vinatzer,B.A., unpublished | PRJNA40341 |
| Pto Max13 | tomato | Vinatzer,B.A., unpublished | PRJNA40343 |
| Pto NCPPB1108 | tomato | Vinatzer,B.A., unpublished | PRJNA40345 |
| Pac A10853 | aceris | This study | PRJNA284850 |
| Pci 0788_9 | cilantroi | This study | PRJNA284851 |
| Pgy BR1 | glycinea | This study | PRJNA284853 |
| Pgy KN44 | glycinea | This study | PRJNA284855 |
| Pgy LN10 | glycinea | This study | PRJNA285209 |
| Pgy UnB647 | glycinea | This study | PRJNA285232 |
| Pla 107 | lachrymans | This study | PRJNA285233 |
| Pla 3988 | lachrymans | This study | PRJNA285235 |
| Pla YM7902 | lachrymans | This study | PRJNA285236 |
| Pma 90_32 | maculicola | This study | PRJNA285237 |
| Pma H7608 | maculicola | This study | PRJNA285238 |
| Pma KN91 | maculicola | This study | PRJNA286818 |
| Pma M4a | maculicola | This study | PRJNA286819 |
| Pma M6 | maculicola | This study | PRJNA286820 |
| Pma YM7930 | maculicola | This study | PRJNA286821 |
| Pme N6801 | mellea | This study | PRJNA287065 |
| Pmp FTRS_U7805 | morsprunorum | This study | PRJNA287067 |
| Pmy AZ84488 | myricae | This study | PRJNA287068 |
| Por 36_1 | oryzae | This study | PRJNA287070 |
| Pph 1302A | phaseolicola | This study | PRJNA287071 |
| Pph HB10Y | phaseolicola | This study | PRJNA287072 |
| Pph NPS3121 | phaseolicola | This study | PRJNA287073 |
| Pph Y5_2 | phaseolicola | This study | PRJNA287074 |
| Psv 4352 | savastanoi | This study | PRJNA287079 |
| Pse HC_1 | sesami | This study | PRJNA287075 |
| Pss A2 | syringae | This study | PRJNA287076 |
| Pss B48 | syringae | This study | PRJNA287078 |

| Table S2 – Predicted Peptide MAMPs ^1^ | | | |
| --- | --- | --- | --- |
| **Gene Description** | **Locus Tag** | **P-value ^2^** | **PSS (#) ^3^** |
| organic solvent tolerance protein, putative | PSPTO_0554 | 2.00E-06 | 23 |
| sucrose-6-phosphate hydrolase | PSPTO_0885 | 3.76E-03 | 16 |
| conserved protein of unknown function | PSPTO_1001 | 5.52E-05 | 14 |
| conserved protein of unknown function | PSPTO_0949 | 1.00E-03 | 14 |
| translation elongation factor Tu | PSPTO_0624 | 3.48E-01 | 14 |
| phospholipase D family protein | PSPTO_1024 | 3.64E-06 | 13 |
| conserved hypothetical protein | PSPTO_0702 | 1.60E-06 | 13 |
| biotin carboxylase/biotin carboxyl carrier protein | PSPTO_5381 | 5.00E-04 | 12 |
| flagellin | PSPTO_1949 | 2.04E-02 | 12 |
| outer membrane protein | PSPTO_1720 | 2.91E-04 | 11 |
| transcriptional regulator, GntR family | PSPTO_5504 | 8.67E-03 | 11 |
| oxidoreductase, FAD-binding protein | PSPTO_0817 | 4.07E-05 | 10 |
| uroporphyrinogen-III synthetase | PSPTO_0129 | 7.00E-03 | 9 |
| macrolide ABC efflux protein | PSPTO_2159 | 1.68E-06 | 9 |
| conserved protein of unknown function | PSPTO_1140 | 2.84E-03 | 9 |
| sigma-54 dependent transcriptional regulator | PSPTO_3144 | 3.20E-03 | 9 |
| lipopolysaccharide core biosynthesis protein WaaG | PSPTO_5001 | 9.30E-03 | 8 |
| conserved hypothetical protein TIGR01125 | PSPTO_4019 | 3.77E-09 | 8 |
| sensory box histidine kinase | PSPTO_0335 | 1.00E-04 | 8 |
| TonB-dependent receptor, putative | PSPTO_1014 | 1.14E-04 | 8 |
| conserved protein of unknown function | PSPTO_4954 | 5.54E-04 | 8 |
| 4-diphosphocytidyl-2C-methyl-D-erythritol kinase | PSPTO_1105 | 3.13E-03 | 8 |
| transcriptional regulator, GntR family | PSPTO_5454 | 7.47E-03 | 8 |
| GGDEF domain protein | PSPTO_2591 | 9.56E-03 | 8 |
| DNA-directed RNA polymerase, beta subunit | PSPTO_0619 | 3.92E-09 | 7 |
| conserved protein of unknown function | PSPTO_0932 | 2.02E-07 | 7 |
| membrane protein, putative | PSPTO_3618 | 1.99E-05 | 7 |
| conserved protein of unknown function | PSPTO_3828 | 4.52E-05 | 7 |
| catalase | PSPTO_5263 | 9.38E-05 | 7 |
| sucrose porin precursor | PSPTO_0890 | 1.31E-03 | 7 |
| 2-(5''-triphosphoribosyl)-3'-dephosphocoenzyme-A synthase | PSPTO_5086 | 5.24E-03 | 7 |
| ABC transporter, permease protein | PSPTO_1359 | 8.46E-03 | 7 |
| tabtoxin resistance protein | PSPTO_4893 | 2.75E-03 | 6 |
| xylose operon regluatory protein | PSPTO_3001 | 2.53E-06 | 6 |
| response regulator | PSPTO_5482 | 4.94E-04 | 6 |
| succinylglutamic semialdehyde dehydrogenase | PSPTO_1835 | 8.71E-04 | 6 |
| xanthine/uracil permease family protein | PSPTO_0772 | 1.13E-03 | 6 |
| translation elongation factor G | PSPTO_0623 | 1.92E-03 | 6 |
| oxidoreductase, zinc-binding protein | PSPTO_5231 | 4.13E-03 | 6 |
| smtA protein | PSPTO_0740 | 2.78E-05 | 5 |
| sensor histidine kinase | PSPTO_5398 | 4.02E-05 | 5 |
| methyl-accepting chemotaxis protein | PSPTO_1493 | 5.08E-05 | 5 |
| ATP-dependent protease La domain protein | PSPTO_0737 | 4.20E-04 | 5 |
| molybdopterin-guanine dinucleotide biosynthesis protein A | PSPTO_2351 | 8.29E-04 | 5 |
| conserved hypothetical protein | PSPTO_5089 | 1.44E-03 | 5 |
| fatty acid/phospholipid synthesis protein PlsX | PSPTO_3834 | 4.67E-03 | 5 |
| D-galactose 1-dehydrogenase | PSPTO_4782 | 9.67E-03 | 5 |
| dnaK protein | PSPTO_4505 | 2.74E-10 | 4 |
| hydroxydechloroatrazine ethylaminohydrolase | PSPTO_0765 | 1.37E-06 | 4 |
| phenylalanine hydroxylase transcriptional activator PhhR | PSPTO_1823 | 2.72E-06 | 4 |
| transcriptional regulator, LysR family | PSPTO_1887 | 6.46E-05 | 4 |
| conserved protein of unknown function | PSPTO_4946 | 1.93E-03 | 4 |
| cobalamin synthesis protein/P47K family protein | PSPTO_4636 | 3.80E-09 | 3 |
| tRNA (uracil-5-)-methyltransferase | PSPTO_4654 | 6.82E-05 | 3 |
| L-arabinose ABC transporter, ATP-binding protein | PSPTO_2639 | 3.73E-04 | 3 |
| D-erythrose 4-phosphate dehydrogenase | PSPTO_0386 | 1.46E-03 | 3 |
| Ser/Thr protein phosphatase family protein | PSPTO_1799 | 1.90E-03 | 3 |
| isochorismatase family protein | PSPTO_1156 | 4.15E-03 | 3 |
| transcriptional regulator, LysR family | PSPTO_1787 | 4.30E-03 | 3 |
| conserved protein of unknown function | PSPTO_1609 | 5.10E-03 | 3 |
| septum site-determining protein MinD | PSPTO_3873 | 8.19E-03 | 3 |
| ^1^ This table includes only those candidates with at least one cluster of at least 3 positively selected sites (using a cluster size of 25 aa). The full table is available from DSG upon request.  ^2^ P-value rejecting null hypothesis of neutral evolution for the protein overall.  ^3^ Number of positively selected sites. | | | |

| Table S3 – Peptide Sequences | |
| --- | --- |
| **Peptide Name** | **Peptide Sequence** |
| xup25 | LIPEGKVAVTTTQAATERKPLEQPR |
| hyp25 | FEAYRGDEKVSAVLTQLPWTHHLII |
| dmk25 | YGDELGFALRQDGVIRLQTDVPGVA |
| atp25 | GRIAAWKIRLATTHTQVDKGALHHG |
| omp25 | IDGELTSAVPNRASLGTNDGKVKIK |
| mpp25 | LKPMLQRACDWLESTADGPQHEPWR |

| Table S4 – Negative Control Peptides | | | | |
| --- | --- | --- | --- | --- |
| **Peptide Name** | **Gene Description** | **NCBI Gene ID** | **Peptide Sequence** |  |
| exb25 | TonB system transport protein exbB | [1181677](http://www.ncbi.nlm.nih.gov/gene/1181677) | LLVHDALEEMRLSANSREREGIKER |  |
| fnr25 | Ferredoxin--NADP reductase fnr1 | [1185703](http://www.ncbi.nlm.nih.gov/gene/1185703) | HLEFFSIKVPDGPLTSQLQHLKEGD |  |
| lip25 | Lipoprotein, putative | [1184837](http://www.ncbi.nlm.nih.gov/sites/entrez?db=gene&term=1184837) | GNYALIDVVWTLGLRDAGATAGSKR |  |
| acc25 | Acetyl-CoA carboxylase subunit beta accD | 1185486 | GAIVGERFVRAANYALENRCPMICF |  |
|  | | | | |

| Table S5 – *Arabidopsis thaliana* LRR-RLK genes | | | |
| --- | --- | --- | --- |
| **RefSeq ID** | ***Arabidopsis* Gene ID** | **Protein Name** | **Gene Description** |
| NP_172061 | AT1G05700 |  | LRR^1^ transmembrane protein kinase protein |
| NP_172169 | AT1G06840 |  | LRR protein kinase family protein |
| NP_172235 | AT1G07550 |  | LRR protein kinase family protein |
| NP_172236 | AT1G07560 |  | LRR protein kinase family protein |
| NP_172244 | AT1G07650 |  | LRR transmembrane protein kinase |
| NP_172335 | AT1G08590 |  | LRR receptor-like protein kinase family protein |
| NP_850942 | AT1G09970 | LRR XI-23, RLK7 | LRR receptor-like protein kinase family protein |
| NP_001077512 | AT1G10850 |  | LRR protein kinase family protein |
| NP_172580 | AT1G11130 | SUB, SCM, SRF9 | LRR protein kinase family protein |
| NP_172708 | AT1G12460 |  | LRR protein kinase family protein |
| NP_172891 | AT1G14390 |  | LRR protein kinase family protein |
| NP_173166 | AT1G17230 |  | LRR receptor-like protein kinase family protein |
| NP_173217 | AT1G17750 | PEPR2, AtPEPR2 | PEP1 receptor 2 |
| NP_173869 | AT1G24650 |  | LRR protein kinase family protein |
| NP_564228 | AT1G25320 |  | LRR protein kinase family protein |
| NP_174039 | AT1G27190 |  | LRR protein kinase family protein |
| NP_174166 | AT1G28440 | HSL1 | HAESA-like 1 |
| NP_564335 | AT1G29720 |  | LRR transmembrane protein kinase |
| NP_174266 | AT1G29730 |  | LRR transmembrane protein kinase |
| NP_174267 | AT1G29740 |  | LRR transmembrane protein kinase |
| NP_850955 | AT1G29750 | RKF1 | Receptor-like kinase in flowers 1 |
| NP_174427 | AT1G31420 | FEI1 | LRR protein kinase family protein |
| NP_174673 | AT1G34110 |  | LRR receptor-like protein kinase family protein |
| NP_174683 | AT1G34210 | SERK2, ATSERK2 | Somatic embryogenesis receptor-like kinase 2 |
| NP_174702 | AT1G34420 |  | LRR transmembrane protein kinase family protein |
| NP_564528 | AT1G48480 | RKL1 | Receptor-like kinase 1 |
| NP_175336 | AT1G49100 |  | LRR protein kinase family protein |
| NP_175476 | AT1G50610 |  | LRR protein kinase family protein |
| NP_175590 | AT1G51790 |  | LRR protein kinase family protein |
| NP_175591 | AT1G51800 |  | LRR protein kinase family protein |
| NP_175592 | AT1G51805 |  | LRR protein kinase family protein |
| NP_175593 | AT1G51810 |  | LRR protein kinase family protein |
| NP_175594 | AT1G51820 |  | LRR protein kinase family protein |
| NP_175595 | AT1G51830 |  | LRR protein kinase family protein |
| NP_175597 | AT1G51850 |  | LRR protein kinase family protein |
| NP_175598 | AT1G51860 |  | LRR protein kinase family protein |
| NP_175600 | AT1G51880 | RHS6 | Root hair specific 6 |
| NP_175601 | AT1G51890 |  | LRR protein kinase family protein |
| NP_175603 | AT1G51910 |  | LRR protein kinase family protein |
| NP_175747 | AT1G53420 |  | LRR transmembrane protein kinase |
| NP_175748 | AT1G53430 |  | LRR transmembrane protein kinase |
| NP_175749 | AT1G53440 |  | LRR transmembrane protein kinase |
| NP_175777 | AT1G53730 | SRF6 | STRUBBELIG-receptor family 6 |
| NP_175957 | AT1G55610 | BRL1 | BRI1 like |
| NP_176009 | AT1G56130 |  | LRR transmembrane protein kinase |
| NP_564709 | AT1G56140 |  | LRR transmembrane protein kinase |
| NP_564710 | AT1G56145 |  | LRR transmembrane protein kinase |
| NP_176262 | AT1G60630 |  | LRR protein kinase family protein |
| NP_176483 | AT1G62950 |  | LRR transmembrane protein kinase family protein |
| NP_176532 | AT1G63430 |  | LRR protein kinase family protein |
| NP_176603 | AT1G64210 |  | LRR protein kinase family protein |
| NP_176789 | AT1G66150 | TMK1 | Transmembrane kinase 1 |
| NP_176855 | AT1G66830 |  | LRR protein kinase family protein |
| NP_176918 | AT1G67510 |  | LRR protein kinase family protein |
| NP_564904 | AT1G67720 |  | LRR protein kinase family protein |
| NP_177087 | AT1G69270 | RPK1 | Receptor-like protein kinase 1 |
| NP_177157 | AT1G69990 |  | LRR protein kinase family protein |
| NP_177328 | AT1G71830 | SERK1, ATSERK1 | Somatic embryogenesis receptor-like kinase 1 |
| NP_177363 | AT1G72180 |  | LRR receptor-like protein kinase family protein |
| NP_177374 | AT1G72300 |  | LRR receptor-like protein kinase family protein |
| NP_177390 | AT1G72460 |  | LRR protein kinase family protein |
| NP_177451 | AT1G73080 | PEPR1, ATPEPR1 | PEP1 receptor 1 |
| NP_565084 | AT1G74360 |  | LRR protein kinase family protein |
| NP_177694 | AT1G75640 |  | LRR receptor-like protein kinase family protein |
| NP_177710 | AT1G75820 | CLV1, FAS3, FLO5, ATCLV1 | LRR receptor-like protein kinase family protein |
| NP_178019 | AT1G78980 | SRF5 | STRUBBELIG-receptor family 5 |
| NP_178080 | AT1G79620 |  | LRR protein kinase family protein |
| NP_178230 | AT2G01210 |  | LRR protein kinase family protein |
| NP_178291 | AT2G01820 |  | LRR protein kinase family protein |
| NP_178304 | AT2G01950 | VH1, BRL2 | BRI1-like 2 |
| NP_178330 | AT2G02220 | ATPSKR1, PSKR1 | Phytosulfokin receptor 1 |
| NP_178381 | AT2G02780 |  | LRR protein kinase family protein |
| NP_178510 | AT2G04300 |  | LRR protein kinase family protein |
| NP_178721 | AT2G07040 | ATPRK2A, PRK2A | LRR protein kinase family protein |
| NP_178999 | AT2G13790 | ATSERK4, SERK4, BKK1, BAK7 | Somatic embryogenesis receptor-like kinase 4 |
| NP_179051 | AT2G14440 |  | LRR protein kinase family protein |
| NP_179057 | AT2G14510 |  | LRR protein kinase family protein |
| NP_179132 | AT2G15300 |  | LRR protein kinase family protein |
| NP_179220 | AT2G16250 |  | LRR protein kinase family protein |
| NP_179509 | AT2G19190 | FRK1 | FLG22-induced receptor-like kinase 1 |
| NP_179511 | AT2G19210 |  | LRR transmembrane protein kinase protein |
| NP_179513 | AT2G19230 |  | LRR transmembrane protein kinase protein |
| NP_565489 | AT2G20850 | SRF1 | STRUBBELIG-receptor family 1 |
| NP_179911 | AT2G23300 |  | LRR protein kinase family protein |
| NP_179973 | AT2G23950 |  | LRR protein kinase family protein |
| NP_850049 | AT2G24230 |  | LRR protein kinase family protein |
| NP_180150 | AT2G25790 |  | LRR receptor-like protein kinase family protein |
| NP_180201 | AT2G26330 | ER, QRP1 | LRR receptor-like protein kinase family protein |
| NP_180241 | AT2G26730 |  | LRR protein kinase family protein |
| NP_180274 | AT2G27060 |  | LRR protein kinase family protein |
| NP_180462 | AT2G28960 |  | LRR protein kinase family protein |
| NP_180463 | AT2G28970 |  | LRR protein kinase family protein |
| NP_180465 | AT2G28990 |  | LRR protein kinase family protein |
| NP_180466 | AT2G29000 |  | LRR protein kinase family protein |
| NP_180747 | AT2G31880 | SOBIR1, EVR | LRR protein kinase family protein |
| NP_180875 | AT2G33170 |  | LRR receptor-like protein kinase family protein |
| NP_181105 | AT2G35620 | FEI2 | LRR protein kinase family protein |
| NP_181196 | AT2G36570 |  | LRR protein kinase family protein |
| NP_181242 | AT2G37050 |  | LRR protein kinase family protein |
| NP_181713 | AT2G41820 |  | LRR protein kinase family protein |
| NP_181758 | AT2G42290 |  | LRR protein kinase family protein |
| NP_182059 | AT2G45340 |  | LRR protein kinase family protein |
| NP_186862 | AT3G02130 | RPK2, TOAD2, CLI1 | Receptor-like protein kinase 2 |
| NP_566213 | AT3G03770 |  | LRR protein kinase family protein |
| NP_187480 | AT3G08680 |  | LRR protein kinase family protein |
| NP_566444 | AT3G13065 | SRF4 | STRUBBELIG-receptor family 4 |
| NP_974311 | AT3G14350 | SRF7 | STRUBBELIG-receptor family 7 |
| NP_566589 | AT3G17840 | RLK902 | Receptor-like kinase 902 |
| NP_188604 | AT3G19700 | IKU2 | LRR protein kinase family protein |
| NP_188771 | AT3G21340 |  | LRR protein kinase family protein |
| NP_189017 | AT3G23750 |  | LRR protein kinase family protein |
| NP_189066 | AT3G24240 |  | LRR receptor-like protein kinase family protein |
| NP_189109 | AT3G24660 | TMKL1 | Transmembrane kinase-like 1 |
| NP_974360 | AT3G25560 | NIK2 | NSP-interacting kinase 2 |
| NP_189443 | AT3G28040 |  | LRR receptor-like protein kinase family protein |
| NP_189486 | AT3G28450 |  | LRR protein kinase family protein |
| NP_189874 | AT3G42880 |  | LRR protein kinase family protein |
| NP_190217 | AT3G46330 | MEE39 | LRR protein kinase family protein |
| NP_190218 | AT3G46340 |  | LRR protein kinase family protein |
| NP_190219 | AT3G46350 |  | LRR protein kinase family protein |
| NP_190221 | AT3G46370 |  | LRR protein kinase family protein |
| NP_190224 | AT3G46400 |  | LRR protein kinase family protein |
| NP_190226 | AT3G46420 |  | LRR protein kinase family protein |
| NP_190536 | AT3G49670 | BAM2 | LRR receptor-like protein kinase family protein |
| NP_190592 | AT3G50230 |  | LRR protein kinase family protein |
| NP_190742 | AT3G51740 | IMK2 | Inflorescence meristem receptor-like kinase 2 |
| NP_190927 | AT3G53590 |  | LRR protein kinase family protein |
| NP_191169 | AT3G56100 | MRLK, IMK3 | Meristematic receptor-like kinase |
| NP_191196 | AT3G56370 |  | LRR protein kinase family protein |
| NP_191342 | AT3G57830 |  | LRR protein kinase family protein |
| NP_192248 | AT4G03390 | SRF3 | STRUBBELIG-receptor family 3 |
| NP_192625 | AT4G08850 |  | LRR receptor-like protein kinase family protein |
| NP_192939 | AT4G12020 | WRKY19, ATWRKY19, MAPKKK11, MEKK4 | Protein kinase family protein |
| NP_193599 | AT4G18640 | MRH1 | LRR protein kinase family protein |
| NP_193747 | AT4G20140 | GSO1 | LRR transmembrane protein kinase |
| NP_193760 | AT4G20270 | BAM3 | LRR receptor-like protein kinase family protein |
| NP_193778 | AT4G20450 |  | LRR protein kinase family protein |
| NP_193811 | AT4G20790 |  | LRR protein kinase family protein |
| NP_193826 | AT4G20940 |  | LRR receptor-like protein kinase family protein |
| NP_193944 | AT4G22130 | SRF8 | STRUBBELIG-receptor family 8 |
| NP_194004 | AT4G22730 |  | LRR protein kinase family protein |
| NP_194105 | AT4G23740 |  | LRR protein kinase family protein |
| NP_194578 | AT4G28490 | RLK5, HAE | LRR receptor-like protein kinase family protein |
| NP_194594 | AT4G28650 |  | LRR transmembrane protein kinase family protein |
| NP_001190865 | AT4G29180 | RHS16 | Root hair specific 16 |
| NP_194728 | AT4G29990 |  | LRR transmembrane protein kinase protein |
| NP_194781 | AT4G30520 |  | LRR protein kinase family protein |
| NP_567870 | AT4G31250 |  | LRR protein kinase family protein |
| NP_567920 | AT4G33430 | BAK1 | BRI1-associated receptor kinase |
| NP_567961 | AT4G34220 |  | LRR protein kinase family protein |
| NP_195341 | AT4G36180 |  | LRR receptor-like protein kinase family protein |
| NP_195442 | AT4G37250 |  | LRR protein kinase family protein |
| NP_974713 | AT4G39270 |  | LRR protein kinase family protein |
| NP_195650 | AT4G39400 | BRI1, CBB2, DWF2, BIN1, ATBRI1 | LRR receptor-like protein kinase family protein |
| NP_195809 | AT5G01890 |  | LRR receptor-like protein kinase family protein |
| NP_195815 | AT5G01950 |  | LRR protein kinase family protein |
| NP_196300 | AT5G06820 | SRF2 | STRUBBELIG-receptor family 2 |
| NP_196311 | AT5G06940 |  | LRR receptor-like protein kinase family protein |
| NP_196332 | AT5G07150 |  | LRR protein kinase family protein |
| NP_196335 | AT5G07180 | ERL2 | ERECTA-like 2 |
| NP_196345 | AT5G07280 | EMS1, EXS | LRR transmembrane protein kinase |
| NP_196564 | AT5G10020 |  | LRR receptor-like protein kinase family protein |
| NP_196925 | AT5G14210 |  | LRR protein kinase family protein |
| NP_197104 | AT5G16000 | NIK1 | NSP-interacting kinase 1 |
| NP_197162 | AT5G16590 | LRR1 | LRR protein kinase family protein |
| NP_197192 | AT5G16900 |  | LRR protein kinase family protein |
| NP_197798 | AT5G24100 |  | LRR protein kinase family protein |
| NP_198389 | AT5G35390 |  | LRR protein kinase family protein |
| NP_198561 | AT5G37450 |  | LRR protein kinase family protein |
| NP_198934 | AT5G41180 |  | LRR transmembrane protein kinase family protein |
| NP_199116 | AT5G43020 |  | LRR protein kinase family protein |
| NP_199283 | AT5G44700 | EDA23, GSO2 | LRR transmembrane protein kinase |
| NP_199390 | AT5G45780 |  | LRR protein kinase family protein |
| NP_199392 | AT5G45800 | MEE62 | LRR protein kinase family protein |
| NP_199396 | AT5G45840 |  | LRR protein kinase family protein |
| NP_568696 | AT5G48380 | BIR1 | BAK1-interacting receptor-like kinase 1 |
| NP_199685 | AT5G48740 |  | LRR receptor-like serine/threonine-protein kinase |
| NP_199705 | AT5G48940 |  | LRR transmembrane protein kinase family protein |
| NP_199777 | AT5G49660 |  | LRR transmembrane protein kinase family protein |
| NP_199787 | AT5G49760 |  | LRR protein kinase family protein |
| NP_199788 | AT5G49770 |  | LRR protein kinase family protein |
| NP_199789 | AT5G49780 |  | LRR protein kinase family protein |
| NP_199948 | AT5G51350 |  | LRR transmembrane protein kinase family protein |
| NP_199969 | AT5G51560 |  | LRR protein kinase family protein |
| NP_200144 | AT5G53320 |  | LRR protein kinase family protein |
| NP_200200 | AT5G53890 | PSKR2, AtPSKR2 | Phytosylfokine-alpha receptor 2 |
| NP_200623 | AT5G58150 |  | LRR protein kinase family protein |
| NP_200773 | AT5G59650 |  | LRR protein kinase family protein |
| NP_200774 | AT5G59660 |  | LRR protein kinase family protein |
| NP_200775 | AT5G59670 |  | LRR protein kinase family protein |
| NP_200776 | AT5G59680 |  | LRR protein kinase family protein |
| NP_200956 | AT5G61480 | PXY | LRR protein kinase family protein |
| NP_201029 | AT5G62230 | ERL1 | ERECTA-like 1 |
| NP_201077 | AT5G62710 |  | LRR protein kinase family protein |
| NP_568971 | AT5G63410 |  | LRR protein kinase family protein |
| NP_568977 | AT5G63710 |  | LRR protein kinase family protein |
| NP_201198 | AT5G63930 |  | LRR protein kinase family protein |
| NP_201371 | AT5G65700 | BAM1 | LRR receptor-like protein kinase family protein |
| NP_201372 | AT5G65710 | HSL2 | HAESA-like 2 |
| NP_569046 | AT5G67200 |  | LRR protein kinase family protein |
| NP_201529 | AT5G67280 | RLK | Receptor-like kinase |
| NP_197548 | AT5G20480 | EFR | EF-TU receptor |
| NP_176279 | AT1G60800 | NIK3 | NSP-interacting kinase 3 |
| NP_197965 | AT5G25930 |  | Protein kinase family protein with LRR domain |
| NP_197569 | AT5G20690 |  | LRR protein kinase family protein |
| NP_188102 | AT3G14840 |  | LRR transmembrane protein kinase |
| NP_201327 | AT5G65240 |  | LRR protein kinase family protein |
| NP_186938 | AT3G02880 |  | LRR protein kinase family protein |
| NP_199445 | AT5G46330 | FLS2 | LRR receptor-like protein kinase family protein |
| NP_171917 | AT1G04210 |  | LRR protein kinase family protein |
| NP_187946 | AT3G13380 | BRL3 | BRI1-like 3 |
| NP_179000 | AT2G13800 | ATSERK5, SERK5, BAK8 | Somatic embryogenesis receptor-like kinase 5 |
| NP_196135 | AT5G05160 |  | LRR protein kinase family protein |
| NP_567748 | AT4G26540 |  | LRR receptor-like protein kinase family protein |
| NP_200415 | AT5G56040 |  | LRR receptor-like protein kinase family protein |
| NP_179990 | AT2G24130 |  | LRR receptor-like protein kinase family protein |
| NP_190293 | AT3G47090 |  | LRR protein kinase family protein |
| NP_566892 | AT3G47570 |  | LRR protein kinase family protein |
| NP_190342 | AT3G47580 |  | LRR protein kinase family protein |
| NP_198755 | AT5G39390 |  | LRR protein kinase-like protein |
| NP_177007 | AT1G68400 |  | LRR transmembrane protein kinase family protein |
| NP_190295 | AT3G47110 |  | LRR protein kinase family protein |
| NP_176008 | AT1G56120 |  | LRR transmembrane protein kinase |
| NP_174809 | AT1G35710 |  | Protein kinase family protein with LRR domain |
| NP_196591 | AT5G10290 |  | LRR transmembrane protein kinase family protein |
| NP_200638 | AT5G58300 |  | LRR protein kinase family protein |
| NP_188654 | AT3G20190 |  | LRR protein kinase family protein |
| ^1^ LRR = Leucine-rich repeat | | | |

1. Almeida NF, Yan S, Lindeberg M, Studholme DJ, Schneider DJ, Condon B, et al. A draft genome sequence of Pseudomonas syringae pv. tomato T1 reveals a type III effector repertoire significantly divergent from that of Pseudomonas syringae pv. tomato DC3000. Mol. Plant. Microbe. Interact. 2009;22:52–62.

2. Baltrus DA, Nishimura MT, Romanchuk A, Chang JH, Mukhtar MS, Cherkis K, et al. Dynamic evolution of pathogenicity revealed by sequencing and comparative genomics of 19 Pseudomonas syringae isolates. PLoS Pathog. 2011;7:e1002132.

3. O’Brien HE, Thakur S, Gong Y, Fung P, Zhang J, Yuan L, et al. Extensive remodeling of the Pseudomonas syringae pv. avellanae type III secretome associated with two independent host shifts onto hazelnut. BMC Microbiol. 2012;12:141.

4. Buell CR, Joardar V, Lindeberg M, Selengut J, Paulsen IT, Gwinn ML, et al. The complete genome sequence of the Arabidopsis and tomato pathogen Pseudomonas syringae pv. tomato DC3000. Proc. Natl. Acad. Sci. U. S. A. 2003;100:10181–6.

5. Feil H, Feil WS, Chain P, Larimer F, DiBartolo G, Copeland A, et al. Comparison of the complete genome sequences of Pseudomonas syringae pv. syringae B728a and pv. tomato DC3000. Proc. Natl. Acad. Sci. U. S. A. 2005;102:11064–9.

6. Green S, Studholme DJ, Laue BE, Dorati F, Lovell H, Arnold D, et al. Comparative genome analysis provides insights into the evolution and adaptation of Pseudomonas syringae pv. aesculi on Aesculus hippocastanum. PLoS One. 2010;5:e10224.

7. Joardar V, Lindeberg M, Jackson RW, Selengut J, Dodson R, Brinkac LM, et al. Whole-genome sequence analysis of Pseudomonas syringae pv. phaseolicola 1448A reveals divergence among pathovars in genes involved in virulence and transposition. J. Bacteriol. 2005;187:6488–98.

8. Scortichini M, Marcelletti S, Ferrante P, Firrao G. A Genomic redefinition of Pseudomonas avellanae species. PLoS One. 2013;8:e75794.

9. Qi M, Wang D, Bradley CA, Zhao Y. Genome sequence analyses of Pseudomonas savastanoi pv. glycinea and subtractive hybridization-based comparative genomics with nine pseudomonads. PLoS One. 2011;6:e16451.

10. Rodríguez-Palenzuela P, Matas IM, Murillo J, López-Solanilla E, Bardaji L, Pérez-Martínez I, et al. Annotation and overview of the Pseudomonas savastanoi pv. savastanoi NCPPB 3335 draft genome reveals the virulence gene complement of a tumour-inducing pathogen of woody hosts. Environ. Microbiol. 2010;12:1604–20.

11. Studholme DJ, Ibanez SG, MacLean D, Dangl JL, Chang JH, Rathjen JP. A draft genome sequence and functional screen reveals the repertoire of type III secreted proteins of Pseudomonas syringae pathovar tabaci 11528. BMC Genomics. 2009;10:395.
