# Supplementary figures and images for: Genomic screens identify a new phytobacterial microbe-associated molecular pattern and the cognate Arabidopsis receptor-like kinase that mediates its immune elicitation
Source: Genome Biol. 2016 May 9;17:98. doi: 10.1186/s13059-016-0955-7 (PMC4862170; doi:10.1186/s13059-016-0955-7)

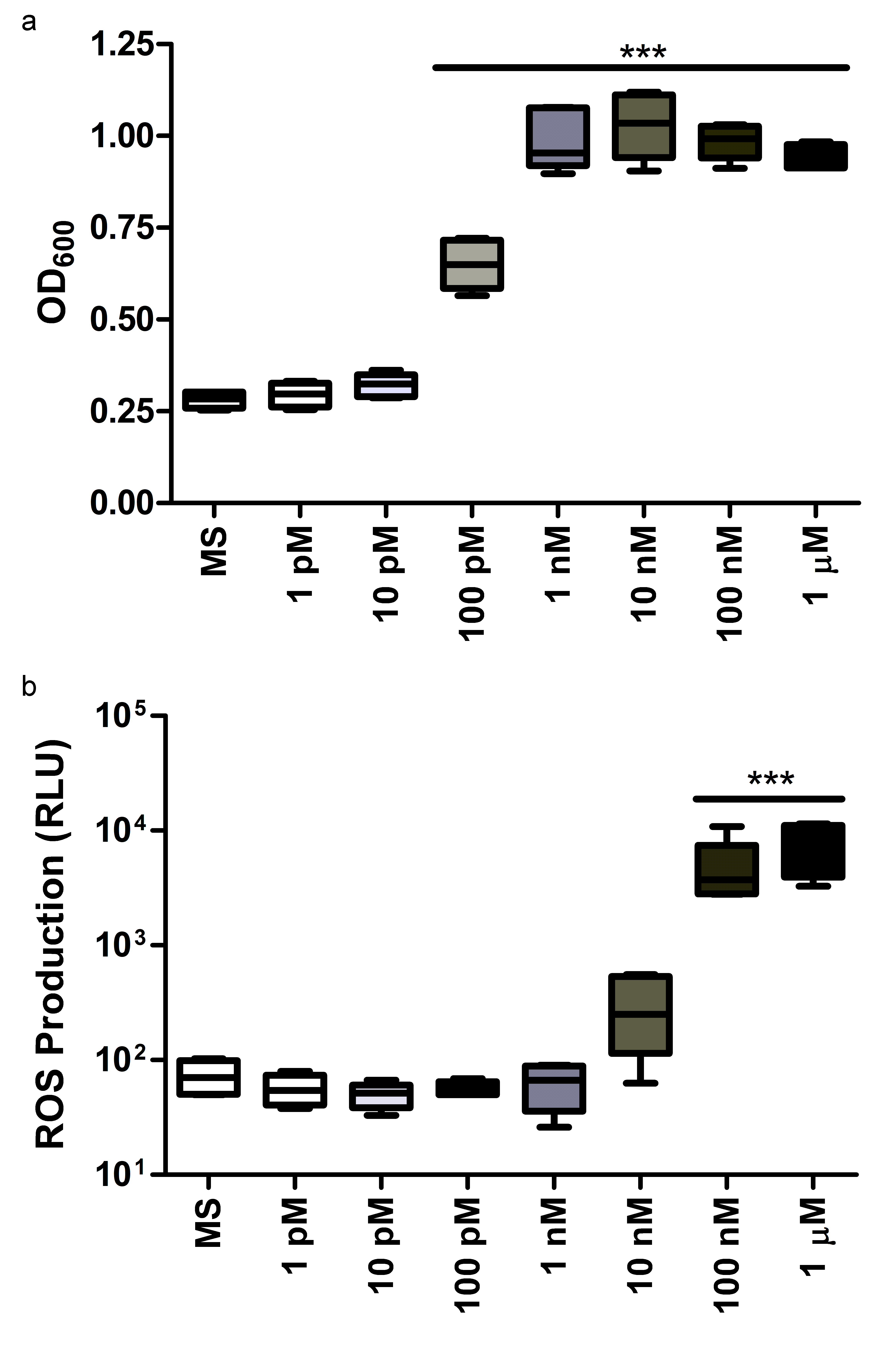

Supplement: Additional file 2: Figure S1. — Comparison of peroxidase- and luminol-based PTI assays. Leaf disks from A. thaliana ecotype Col-0 plants were treated with water or the noted dose of flg22 peptide. Graphs are data from a single representative experiment. a Total POX activity was measured 20 h after treatment (n = 6, ***P <0.001, pairwise Student’s t-test, corrected with Holm-Bonferroni). b ROS production was quantified using luminol and horseradish peroxidase with luminescence measured for 2 s every 2 min for 60 min and the total summed. (n = 6, ***P <0.001, pairwise Student’s t-test, corrected with Holm-Bonferroni). Boxes show the lower quartile value, median value, and upper quartile value, while the whiskers extend to the lowest and highest values. (PNG 216 kb) [file 13059_2016_955_MOESM2_ESM.png]

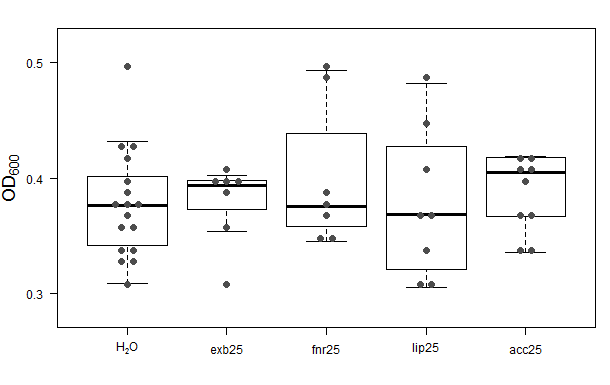

Supplement: Additional file 3: Figure S2. — Negative control peptides without predicted positively selected sites do not cause increased POX activity. Leaf disks from A. thaliana ecotype Col-0 plants were treated with water or 1 μM of the indicated negative control peptide and total POX activity was measured 20 h after treatment. The mean value was calculated for each experimental replicate (n = 6) performed and these means plotted as data points on the graph. Each peptide was tested independently eight or nine times. Boxplot layout is as described in Fig. 1. (PNG 3 kb) [file 13059_2016_955_MOESM3_ESM.png]

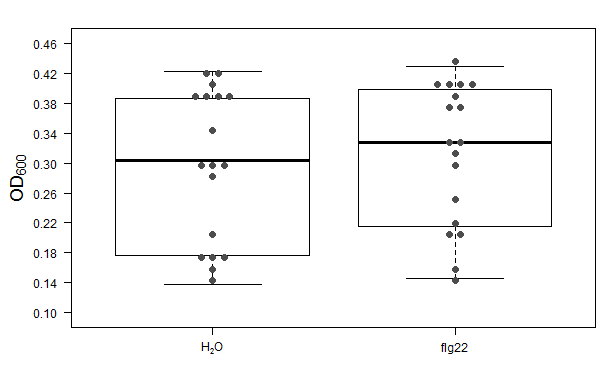

Supplement: Additional file 4: Figure S3. — flg22 does not cause increased POX activity in Arabidopsis ecotype Ws. Leaf disks from A. thaliana ecotype Ws plants were treated with water or 1 μM of flg22 peptide. Total POX activity was measured 20 h after treatment. The experiment was replicated three times for a total n = 18. Boxplot layout is as described in Fig. 1. (PNG 3 kb) [file 13059_2016_955_MOESM4_ESM.png]

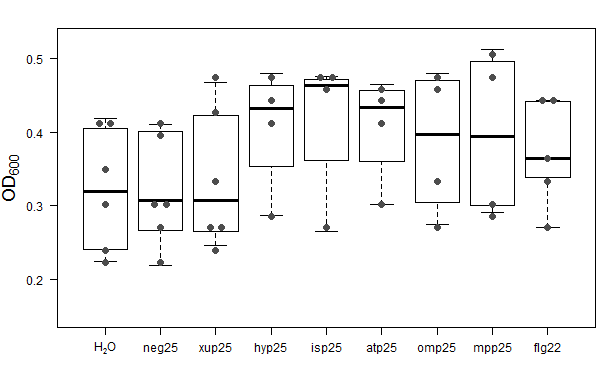

Supplement: Additional file 5: Figure S4. — xup25 does not induce POX activity in an independent xps1 T-DNA insertion line: xps1-2. Leaf disks from A. thaliana xps1-2 plants were treated with water or 1 μM of peptide and total POX activity was measured 20 h after treatment. The mean value was calculated for each experimental replicate (n = 6) performed and these means plotted as data points on the graph. Each peptide was tested a total of four times except xup25 which was tested six times. Boxplot layout is as described in Fig. 1. (PNG 4 kb) [file 13059_2016_955_MOESM5_ESM.png]

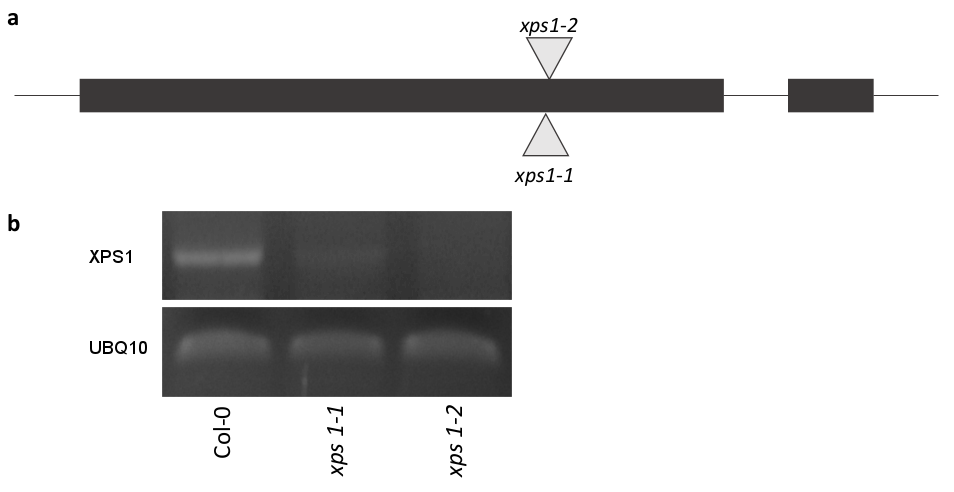

Supplement: Additional file 6: Figure S5. — Confirmation of T-DNA insertions for two independent xps1 T-DNA insertion lines. a T-DNA insertion position for xps1-1 and xps1-2. Boxes represent exons and lines represent introns. b RT-PCR analysis of XPS1 expression in Col-0, xps1-1, and xps1-2 plants, respectively. The ubiquitously expressed UBQ10 is shown as a control for cDNA concentration. (PNG 51 kb) [file 13059_2016_955_MOESM6_ESM.png]

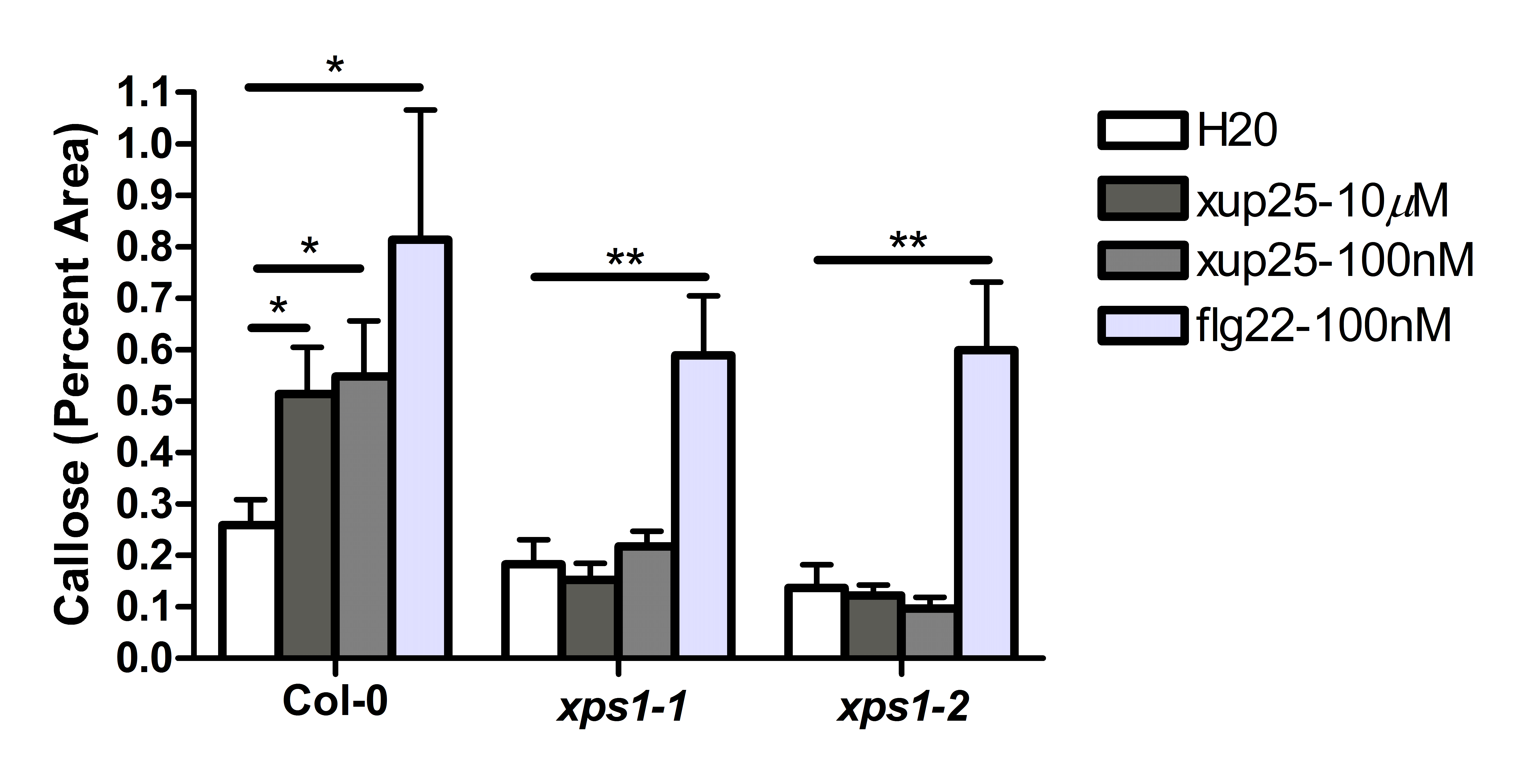

Supplement: Additional file 7: Figure S6. — xup25 does not induce callose deposition in an independent xps1 T-DNA insertion line: xps1-2. Leaves from A. thaliana xps1-2 plants were pressure infiltrated with water or 10 μM of xup25, 100 nM xup25, or flg22 peptide. After 24 h of treatment the leaves were harvested, cleared, and callose deposits were stained prior to epifluorescent microscopy. The proportion of the image with callose present was determined (n = 6, *P <0.05, **P <0.01, pairwise Student’s t-test, corrected with Holm-Bonferroni). No significant difference was observed between Col-0, xps1-1, and xps1-2 plants treated with flg22. (PNG 242 kb) [file 13059_2016_955_MOESM7_ESM.png]

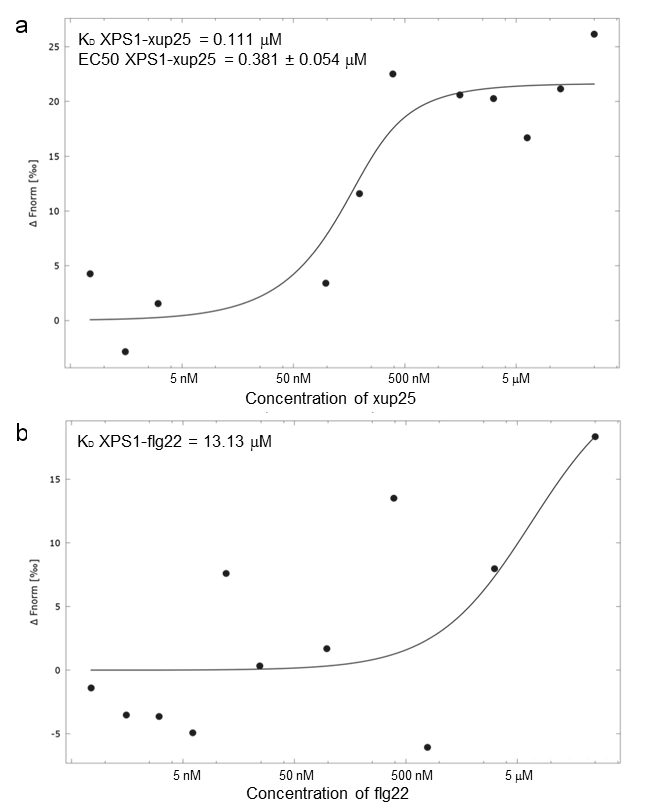

Supplement: Additional file 8: Figure S7. — XPS1 binds specifically to xup25. Additional replicates of XPS1 binding assays. Titration of increasing amounts of xup25 peptide (a), but not flg22 (b), to a constant amount of XPS1 ECD (0.5 μM) induces a significant MST signal shift. At each peptide concentration a measurement was made using 60 % MST power and the values used to determine the KD or the EC50 with the NT affinity analysis software from Nanotemper. The KD fit plots are shown and both the KD and EC50 values are noted wherever possible. The calculated EC50 and KD values for xup25 were EC50 of 0.381 μM and KD of 0.111 μM. The flg22 binding data could not be fitted using the EC50 fit and has a KD of 13.13 μM. The standard error of the regression fit is 3.8404566 for (a) and 6.4503735 for (b). (PNG 20 kb) [file 13059_2016_955_MOESM8_ESM.png]

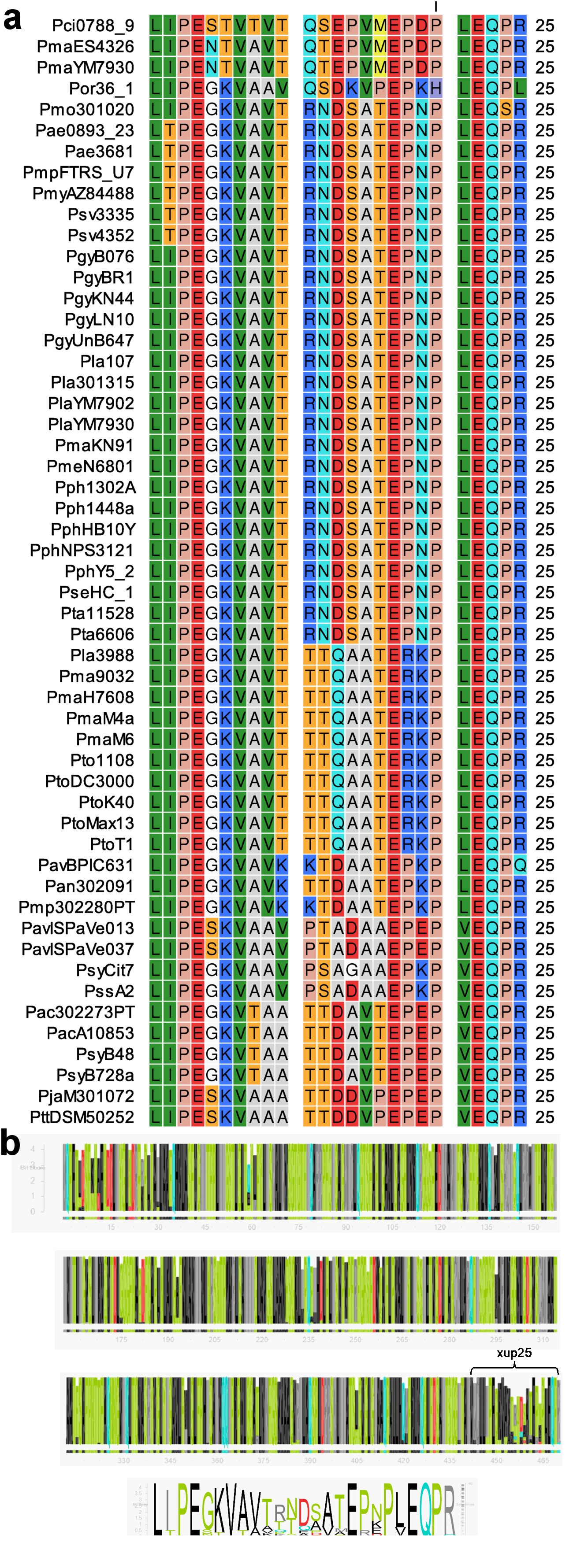

Supplement: Additional file 9: Figure S8. — Diversity of xup25 sequence across P. syringae species. a The protein sequences of the xup25 peptide from each species used in this study aligned. b The protein sequence of the xanthine uracil permease gene was aligned and used to produce a sequence logo, the xup25 sequence logo is marked and expanded below showing the diversity present in the peptide sequence. (PNG 1736 kb) [file 13059_2016_955_MOESM9_ESM.png]
